# B.R.E.A.S.T. Breast canceR Enhanced AI-Supported Therapy: A New Interpretable Proteomics-Driven Machine Learning Framework for Therapy Response Prediction in Breast Cancer

**DOI:** 10.3390/ijms27125163

**Published:** 2026-06-06

**Authors:** Alessia Bono, Gabriele La Monica, Federica Alamia, Dennis Tocco, Antonino Lauria, Annamaria Martorana

**Affiliations:** 1 Dipartimento di Scienze e Tecnologie Biologiche Chimiche e Farmaceutiche “STEBICEF”, University of Palermo, Viale delle Scienze, Ed. 17, 90128 Palermo, Italy; alessia.bono01@unipa.it (A.B.); gabriele.lamonica01@unipa.it (G.L.M.); federica.alamia01@unipa.it (F.A.); dennis.tocco@unipa.it (D.T.); annamaria.martorana@unipa.it (A.M.); 2 Fondazione Umberto Veronesi (FUV), Via Solferino 19, 20121 Milano, Italy

**Keywords:** breast cancer, artificial intelligence, machine learning, proteomics, personalized therapy, biomarker discovery, SHAP

## Abstract

Breast cancer is a heterogeneous disease characterized by substantial molecular diversity and variable treatment outcomes across patients. Despite advances in targeted and systemic therapies, anticipating individual benefit remains a major clinical challenge. In this context, Artificial Intelligence (AI) can support precision oncology by integrating high-dimensional molecular profiles with clinical and pharmacological information. Here, we present B.R.E.A.S.T. (Breast canceR Enhanced AI-Supported Therapy), an interpretable machine learning framework designed to predict therapy outcome from tumor proteomic profiles integrated with clinical and treatment annotations. Proteomic data from The Cancer Genome Atlas (TCGA) and The Cancer Proteome Atlas (TCPA) were harmonized with outcome and therapy information, and thirteen supervised classifiers were systematically evaluated using stratified 5-fold cross-validation. Therapeutic outcome labels were operationally defined by integrating available treatment response annotations with complementary clinical outcome information. Across both cohorts, ensemble-based models consistently achieved the most stable and highest discriminative performance, supported by learning-curve analyses and consistent behavior across independent datasets. To enhance interpretability, we implemented a two-step feature selection strategy combining model-specific importance measures with a global consensus ranking, enabling the identification of a compact set of robust proteomic biomarkers associated with therapeutic outcome. Top-ranked features mapped to molecular programs relevant to breast cancer progression and treatment sensitivity, including regulators of cell survival, DNA damage response, PI3K/AKT/mTOR signaling, and invasion-related processes. Re-evaluation using only the top 30 globally ranked features preserved high predictive performance across both independent breast cancer cohorts, indicating that a parsimonious proteomic signature captures core molecular determinants of outcome. Overall, B.R.E.A.S.T. provides a robust and generalizable proteomics-driven framework for modeling outcome-associated therapeutic response patterns and supporting biologically informed biomarker discovery in breast cancer.

## 1. Introduction

Artificial Intelligence (AI) and machine learning (ML) are rapidly transforming biomedical research and clinical decision-making. By enabling the integration and analysis of high-dimensional molecular datasets, ML approaches provide powerful tools for uncovering complex, non-linear relationships that may underlie disease progression and therapeutic response. In oncology, these methodologies have demonstrated considerable promise in tumor classification, prognosis prediction, radiogenomics, and drug response modeling [1,2,3,4,5]. However, despite increasing methodological sophistication, several critical challenges remain. Many predictive models lack robust cross-cohort validation, rely on limited algorithmic benchmarking, or operate as “black-box” systems with insufficient biological interpretability [6]. Consequently, the clinical translation of ML-based predictions remains constrained.

A central unmet need in precision oncology is the development of predictive frameworks that combine strong discriminative performance with mechanistic transparency. While high predictive accuracy is essential, clinical adoption also requires models to provide interpretable and biologically coherent explanations linking molecular features to treatment response. Bridging predictive modeling with pathway-level understanding therefore represents a key methodological priority.

Breast cancer (BC) constitutes a particularly relevant context in which to address these challenges. Despite advances in screening programs, targeted therapies, and systemic treatment strategies, substantial interpatient variability in therapeutic response and the emergence of resistance continue to limit clinical outcomes in a significant subset of patients [7]. This variability reflects the marked molecular heterogeneity of BC, which encompasses distinct genomic alterations, signaling network configurations, and pathway activation states.

Although genomic and transcriptomic profiling have considerably advanced molecular classification, proteins ultimately function as the primary effectors of cellular signaling networks. Post-translational modifications, kinase activation states, and dynamic pathway interactions are not fully captured by DNA- or RNA-based analyses. Proteomic profiling therefore provides a direct functional readout of tumor biology and may offer enhanced resolution for modeling therapeutic sensitivity and resistance mechanisms [8,9].

Several prior studies have applied machine learning approaches to cancer stratification, biomarker discovery, or subtype classification [10,11,12,13,14,15,16,17,18,19]. While these investigations provide valuable insights, important methodological limitations persist. Some studies incorporated biologically informed modeling strategies [10] or ensemble learning approaches targeting specific pathways [11], whereas others focused on multi-omics integration or subtype assignment [12,15,17]. However, many were not specifically designed to predict therapeutic response using proteomic data, relied on limited cohort sizes, or evaluated a restricted number of algorithms without systematic benchmarking. Cross-dataset validation was frequently absent, limiting assessment of model generalizability. Concerns regarding reproducibility, overfitting, and inadequate validation strategies in biomedical machine learning have been widely discussed in the literature [20]. Moreover, interpretability analyses were often confined to feature ranking without integrating pathway-level contextualization or independent directional validation of response-associated expression differences. Although conceptual frameworks for drug prioritization and AI validation in precision medicine have been proposed [18,19], comprehensive proteomics-driven, cross-cohort-validated response prediction models in breast cancer remain lacking [21].

Large publicly available resources, including The Cancer Genome Atlas (TCGA) [22], The Cancer Proteome Atlas (TCPA) [23,24,25], and cBioPortal [26,27,28], have generated comprehensive molecular and clinical datasets that create unprecedented opportunities for proteomics-driven predictive modeling. The increasing availability of these large-scale, well-annotated datasets enables the development and systematic validation of machine learning frameworks aimed at predicting therapeutic response and supporting precision oncology strategies.

Collectively, these considerations underscore the need for an interpretable, systematically benchmarked, and cross-dataset-validated machine learning framework specifically tailored to proteomic predictors of therapeutic response in breast cancer.

To address this gap, we developed B.R.E.A.S.T. (Breast canceR Enhanced AI-Supported Therapy), an interpretable machine learning framework designed to predict therapeutic response by integrating tumor proteomic profiles with clinical and pharmacological annotations. The framework systematically benchmarks thirteen supervised classification algorithms, implements a two-step consensus-based feature selection strategy, and incorporates SHapley Additive exPlanations (SHAP) to enhance interpretability at both feature and pathway levels.

Importantly, the framework was evaluated across two independent large-scale cohorts (TCGA and TCPA), enabling assessment of cross-dataset robustness and reproducibility. By combining rigorous algorithmic benchmarking, parsimonious feature selection, and biologically grounded interpretability analyses—including directional expression comparisons between Responders and Non-Responders—this study aims to move beyond purely predictive modeling toward mechanistically informed therapy stratification.

Through the integration of large-scale proteomic data, advanced machine learning methodologies, and interpretability-driven validation, B.R.E.A.S.T. establishes a scalable and extensible platform for proteomics-informed precision oncology in breast cancer.

## 2. Results and Discussion

**Figure 1:**
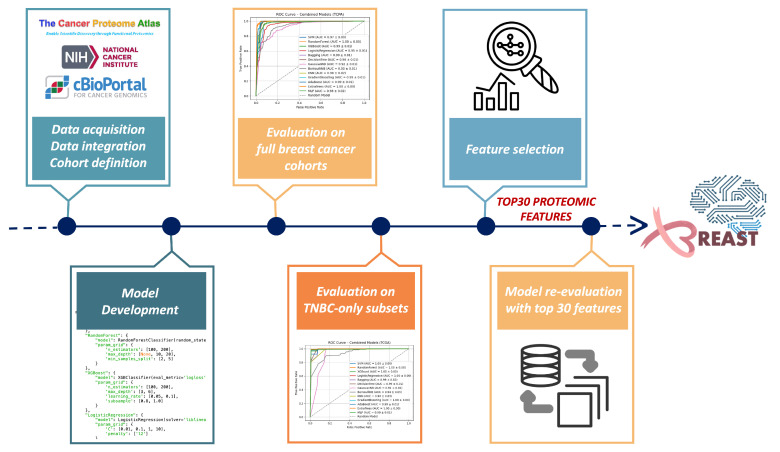
The workflow illustrates the stepwise development and validation of the B.R.E.A.S.T. framework. Proteomic data from TCGA and TCPA are integrated with clinical and therapeutic information from cBioPortal, followed by supervised machine learning model development and evaluation on full breast cancer cohorts. A global feature selection strategy identifies the top 30 proteomic features, which are subsequently used for reduced-feature modeling and re-evaluation. The framework ultimately enables robust and interpretable prediction of therapeutic response and the identification of biologically relevant proteomic biomarkers in Breast Cancer.

This study proposes a structured and data-driven workflow for the development of a machine learning-based AI framework capable of predicting therapeutic response based on Breast Cancer proteomic profiles. The overall workflow of the B.R.E.A.S.T. framework is illustrated in Figure 1 and is organized into a sequence of interconnected steps designed to ensure robustness, interpretability, and predictive accuracy.

The first phase of the workflow focused on data collection, integration, and matching. Proteomic data were derived from TCGA and TCPA, while clinical and therapeutic information was obtained from cBioPortal. This integration process resulted in a curated dataset suitable for downstream machine learning analyses and therapy-response modelling. In the second phase, the integrated dataset was used for model development. Multiple machine learning algorithms were systematically evaluated to identify the most effective predictive strategy. Model training was performed using a 5-fold cross-validation scheme to reduce overfitting and ensure generalizability. In the third phase, model performance was evaluated on the breast cancer cohorts derived from TCGA and TCPA. Predictive performance was assessed using standard classification metrics, including accuracy, precision, recall, F1-score, and the Area Under the Receiver Operating Characteristic Curve (ROC-AUC), enabling objective comparison among different algorithms.

A feature selection strategy was applied to identify the most informative proteomic biomarkers. Feature importance was first computed independently for each machine learning algorithm, followed by the construction of a global consensus ranking that highlighted features consistently selected across models. Based on this ranking, a reduced set of the top 30 proteomic features was identified. In the final phase of the workflow, model performance was re-evaluated using only the reduced 30-feature set. This step assessed whether a parsimonious and interpretable proteomic signature could preserve high predictive performance across datasets and clinical settings.

### 2.1. Data Collection and Dataset Construction

Data collection and dataset construction represented a critical step in the development of the B.R.E.A.S.T. framework and involved the integration of heterogeneous data sources to obtain a unified, high-quality dataset suitable for machine learning analyses.

Clinical and therapeutic data were retrieved from cBioPortal [26,27,28], while proteomic expression data were obtained from TCGA [22] and TCPA [23,24,25]. The data integration process required multiple preprocessing stages to ensure accurate correspondence between patient identifiers and the associated molecular and clinical information.

Given the heterogeneity and incompleteness typical of large-scale clinical datasets, a systematic strategy was applied to manage missing values. Proteins exhibiting more than 95% missing values across samples were excluded from further analyses, as such features were unlikely to provide reliable predictive information and could introduce noise into machine learning models. As a result, 687 proteins with acceptable data coverage were retained for downstream analyses. Following proteomic filtering, patient-level integration was performed using unique patient/sample identifiers to ensure that each individual included in the dataset possessed consistent proteomic, clinical, and therapeutic information. Patients lacking either pharmacological treatment data or proteomic profiles from both TCGA and TCPA were excluded. This filtering step led to the removal of a limited number of patient identifiers, further refining dataset completeness.

Therapy-response labels were generated by integrating treatment-related and clinical outcome annotations. The treatment_best_response field was used as the primary source of response information when available, as it provides the best clinical response recorded after treatment. This variable was considered conceptually aligned with standard oncological response descriptors, although it was not interpreted as a formal Response Evaluation Criteria in Solid Tumors (RECIST)-based endpoint because RECIST criteria were not explicitly and uniformly available across all patients. When treatment_best_response was missing, unknown, or not informative, response assignment was derived from disease-specific survival status, overall survival status, disease-free status, and the occurrence of new neoplasm events after initial therapy. Accordingly, patients who were alive or deceased but tumor-free were assigned to the Responder class, whereas patients with persistent disease, recurrence/progression, or tumor-related death were assigned to the Non-Responder class.

Pharmacological treatment data underwent an additional normalization step to address inconsistencies in drug nomenclature. Multiple synonymous drug names referring to the same active compound were unified under a standardized label. This harmonization step was essential to prevent artificial fragmentation of therapeutic categories and to ensure meaningful associations between treatments and molecular profiles. Finally, the curated dataset was reorganized into two aligned data matrices corresponding to TCGA- and TCPA-derived proteomic profiles, with consistent clinical, therapeutic, and outcome annotations. Additional patient identifiers lacking complete proteomic data were removed, resulting in the final dataset (see Supplementary Material S1, Tables S1 and S2) used for model development and evaluation within the B.R.E.A.S.T. framework.

### 2.2. Model Development

The development of the machine learning framework was aimed at identifying predictive patterns linking proteomic profiles to therapeutic response in patients with Breast Cancer. Given the high dimensionality of the proteomic data and the intrinsic heterogeneity of clinical outcomes, multiple supervised learning algorithms were systematically explored to ensure robustness and generalizability. The prediction task was formulated as a binary classification problem, in which patients were labeled as “Responders” or “Non-Responders” as described in Section 2.1. The response variable was encoded as a binary outcome, with Responders assigned to class 1 and Non-Responders to class 0, providing a clear and interpretable outcome for model training and evaluation. Proteomic expression features were used as the primary input to the machine learning models and were integrated with selected clinical and therapeutic variables to capture both molecular and treatment-related information relevant to response prediction.

#### 2.2.1. Machine Learning Algorithms

Several supervised machine learning algorithms were implemented and systematically compared to identify the most effective approach for predicting therapeutic response in Breast Cancer patients [29]. In total, thirteen classification algorithms were evaluated, encompassing a wide range of learning paradigms: Support Vector Machine (SVM), Random Forest (RF), XGBoost, Logistic Regression (LGR), Bagging, Decision Tree (DT), Gaussian Naive Bayes (GNB), Bernoulli Naive Bayes (BNB), k-Nearest Neighbors (KNN), Gradient Boosting (GB), AdaBoost, ExtraTrees, and Multilayer Perceptron (MLP) [30,31,32,33].

These models were selected based on their demonstrated complementary capabilities in capturing linear and non-linear relationships [29]. Tree-based ensemble methods, including RF, ExtraTrees, AdaBoost, Bagging, and GB, were employed for their robustness to noise, ability to model complex feature interactions, and resistance to overfitting. SVMs were implemented using nonlinear kernels to identify optimal decision boundaries in high-dimensional feature spaces. Probabilistic classifiers, such as GNB and BNB, were included for their computational efficiency and interpretability. Additionally, kNN was used as a distance-based method sensitive to local data structure, and a MLP neural network was employed to capture complex, nonlinear dependencies within the proteomic profiles. For each algorithm, hyperparameters were optimized using Grid-search procedures within the training folds of the cross-validation framework to maximize predictive performance while minimizing overfitting [34,35].

#### 2.2.2. Data Preprocessing and Feature Engineering

Proteomic expression data derived from Reverse Phase Protein Array (RPPA) profiling were combined with pharmacological treatment information to construct a unified feature space. Drug names were first standardized to resolve inconsistencies and synonymous labels, after which therapies were encoded using a one-hot representation [36]. This strategy enabled explicit modeling of treatment-specific effects while preserving compatibility across ML algorithms. Missing values in numerical features were handled using mean imputation to retain samples and reduce bias introduced by incomplete data. All features were subsequently standardized using z-score normalization to ensure comparable scaling and to optimize the performance of distance- and gradient-based classifiers. The final feature matrix thus consisted of normalized proteomic features and binary indicators of administered therapies.

#### 2.2.3. Cross-Validation Strategy

To ensure reliable performance estimation and reduce sampling bias, a stratified 5-fold cross-validation strategy was adopted. The dataset was randomly partitioned into five equally sized folds while preserving the original class distribution between Responders and Non-Responders. In each iteration, four folds (80% of the data) were used for model training and hyperparameter optimization, while the remaining fold (20%) served as an independent validation set. This procedure was repeated five times, allowing each fold to be used once for validation.

Performance metrics were subsequently averaged across all folds to provide a robust estimate of the model’s generalization capability. Model performance was evaluated using multiple complementary metrics to enable a comprehensive and balanced comparison across classifiers. Accuracy, precision, recall, F1-score, and Receiver Operating Characteristic Area Under the Curve (ROC-AUC) were computed for each model. ROC-AUC was used as a threshold-independent metric to assess discriminative ability across varying classification thresholds, whereas accuracy, precision, recall, and F1-score provided insight into classification correctness and class-specific performance. The ROC curve plots the True Positive Rate (TPR) against the False Positive Rate (FPR) across varying classification thresholds, while the AUC provides a summary measure of the model’s discriminative ability, with values closer to 1 indicating superior performance.

To enhance model interpretability and biological insights, SHapley Additive exPlanations (SHAP) were applied to quantify the contribution of each proteomic feature to the final predictions [37]. This approach enables identification of key biomarkers driving therapeutic response and supports clinical interpretability of the AI framework.

Model selection was based on a combination of predictive performance, stability across cross-validation folds, and interpretability. Rather than prioritizing a single performance metric, models were evaluated holistically to identify the approach that best balanced accuracy, robustness, and clinical relevance.

The best-performing model was subsequently selected for downstream evaluation and biological interpretation, as described in the following sections.

#### 2.2.4. Overall Performance of Machine Learning Models

The performance of thirteen supervised machine learning algorithms was systematically evaluated on two independent proteomic datasets, TCGA and TCPA. Model performance was assessed primarily through ROC-AUC, complemented by accuracy, precision, recall, and F1 score to provide a comprehensive evaluation of classification quality and stability across folds. The optimal hyperparameter configuration was identified for each machine learning algorithm. For each classifier, the ROC-AUC was calculated using the best-performing hyperparameter combination, reflecting the discriminative performance achieved under optimized conditions. Heatmap representations of these ROC-AUC values for all algorithms and datasets are reported in the Supplementary Material S2, Figures S1–S22 and summarized in Table S3.

Across both datasets, ensemble-based methods consistently demonstrated superior predictive performance, achieving high discriminative power and low variability, while simpler probabilistic models showed reduced robustness in distinguishing Responders from Non-Responders.

### 2.3. Performance on the TCGA Dataset

On the TCGA cohort, the majority of ML models achieved excellent predictive performance, further confirming the robustness of the proposed framework across independent proteomic datasets. As reported in Table 1, most algorithms reached mean accuracy values above 0.98, with ROC-AUC scores consistently exceeding 0.98 for advanced classifiers, indicating a strong ability to discriminate between Responder and Non-Responder Breast Cancer patients. The results were obtained using Script S1 in Supplementary Material S3.

On the TCGA dataset, ensemble-based methods demonstrated the best overall performance. Random Forest, Gradient Boosting, AdaBoost, Bagging, and ExtraTrees classifiers achieved near-perfect classification results, with mean accuracies of approximately 0.996 and F1-scores close to 0.998. Notably, the ExtraTrees model emerged as the top-performing algorithm, achieving the highest discriminative power with a mean ROC-AUC of 0.999 ± 0.001, alongside perfect recall (1.000 ± 0.000). This indicates an exceptional capability to correctly identify Responder patients while minimizing false-negative predictions, a critical aspect in a clinical decision-support context. Gradient Boosting and Random Forest models also exhibited outstanding stability and robustness, both achieving ROC-AUC values close to 0.997 with minimal variability across cross-validation folds. These results highlight the effectiveness of tree-based ensemble approaches in capturing complex nonlinear interactions within high-dimensional proteomic data derived from TCGA. XGBoost further confirmed the suitability of boosting strategies, achieving a mean ROC-AUC of 0.993 ± 0.006 and an F1-score of 0.994 ± 0.004, reinforcing its ability to model intricate feature relationships relevant to therapeutic response. Support Vector Machines achieved strong and consistent performance, with a mean ROC-AUC of 0.997 ± 0.002 and an F1-score of 0.991 ± 0.003, indicating that nonlinear kernel-based decision boundaries are well suited to the structure of TCGA proteomic profiles. Similarly, k-Nearest Neighbors demonstrated high predictive accuracy (0.993 ± 0.004) and a ROC-AUC of 0.989 ± 0.011, suggesting that locally similar proteomic patterns are informative of treatment response. In contrast, probabilistic classifiers exhibited inferior performance compared to ensemble and kernel-based methods. Gaussian Naive Bayes showed markedly reduced accuracy (0.589 ± 0.020) and recall (0.527 ± 0.022), despite maintaining high precision, likely reflecting the strong violation of conditional independence assumptions in complex proteomic datasets. Bernoulli Naive Bayes also underperformed relative to other models, achieving a ROC-AUC of 0.858 ± 0.018, further emphasizing the limitations of simplified probabilistic approaches in this context. Overall, the TCGA results indicate that ensemble-based models provide highly robust and stable predictive performance. In particular, ExtraTrees, Random Forest, and Gradient Boosting emerged as the most reliable algorithms and were therefore considered suitable candidates for downstream SHAP-based interpretability analyses and biological interpretation. These findings further support the reliability of proteomics-driven ML approaches for predicting therapeutic response in Breast Cancer patients.

**Figure 2:**
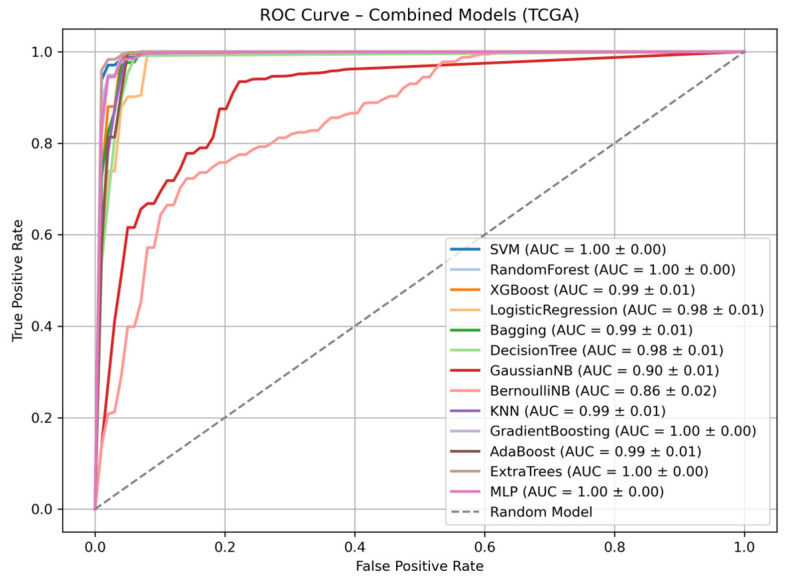
ROC curves for all thirteen supervised ML models applied to the TCGA dataset.

Figure 2 illustrates the ROC curves obtained from the stratified 5-fold cross-validation for the thirteen machine learning models evaluated on the TCGA dataset, highlighting their discriminative performance in distinguishing Responder from Non-Responder patients.

### 2.4. Performance on the TCPA Dataset

On the TCPA cohort, most ML models achieved excellent classification performance, with ROC-AUC values exceeding 0.97 for the majority of algorithms. Table 2 reports the predictive performance of the thirteen supervised ML models evaluated on the TCPA dataset, as assessed by stratified 5-fold cross-validation. For each algorithm, the table presents the mean ± standard deviation of accuracy, precision, recall, F1-score, and ROC-AUC (all the results have been obtained through Script S1 in Supplementary Material S3).

In line with the results obtained on the TCGA cohort, ensemble-based methods consistently outperformed simpler classifiers, demonstrating remarkable accuracy, robustness, and stability across cross-validation folds. Random Forest, Gradient Boosting, ExtraTrees, Bagging, and AdaBoost achieved near-perfect classification performance, with mean accuracy values exceeding 0.99 and ROC-AUC values ranging between 0.987 and 0.998. In particular, Random Forest and ExtraTrees emerged as the top-performing models, both achieving a mean ROC-AUC of 0.998 ± 0.002, accompanied by perfect or near-perfect recall (1.000 ± 0.000), indicating an exceptional ability to correctly identify Responder patients while minimizing false negatives.

XGBoost also demonstrated strong predictive capabilities, achieving a mean ROC-AUC of 0.988 ± 0.010 and an F1-score of 0.995 ± 0.002, confirming the effectiveness of gradient-based boosting strategies in modeling complex nonlinear relationships within high-dimensional proteomic data. Similarly, Gradient Boosting and AdaBoost classifiers achieved highly competitive results, with ROC-AUC values above 0.99 and balanced precision–recall profiles, further reinforcing the suitability of ensemble learning approaches for this task.

Support Vector Machines with nonlinear kernels achieved robust performance, reaching a mean ROC-AUC of 0.970 ± 0.003 and an F1-score of 0.982 ± 0.003. Although slightly inferior to ensemble-based methods, SVMs maintained high recall (0.987 ± 0.005), suggesting reliable sensitivity in detecting Responder patients. Multilayer Perceptron neural networks exhibited comparable performance (ROC-AUC = 0.975 ± 0.017), indicating that nonlinear neural architectures can effectively capture complex proteomic patterns, albeit with slightly higher variability across folds.

Distance-based and probabilistic models displayed more heterogeneous behavior. k-Nearest Neighbors achieved strong overall performance (ROC-AUC = 0.979 ± 0.017), particularly when using distance-weighted voting and Manhattan distance, whereas Logistic Regression showed comparatively lower discriminative ability (ROC-AUC = 0.953 ± 0.015), reflecting the limitations of linear decision boundaries in capturing the intrinsic complexity of proteomic data. Gaussian Naive Bayes achieved high precision but suffered from substantially reduced recall (0.685 ± 0.030), resulting in lower overall accuracy and F1-score, likely due to the violation of feature independence assumptions in high-dimensional proteomic datasets.

Collectively, these results demonstrate that ensemble-based machine learning models provide superior and highly stable performance in predicting therapeutic response in Breast Cancer patients using TCPA proteomic data. The consistently high ROC-AUC values and minimal variability across cross-validation folds underscore the robustness of the proposed framework and support the biological relevance of the selected features. Based on this comprehensive comparative analysis, ensemble models, particularly Random Forest and ExtraTrees, were identified as the most reliable candidates for downstream biological interpretation and SHAP-based feature attribution analyses.

**Figure 3:**
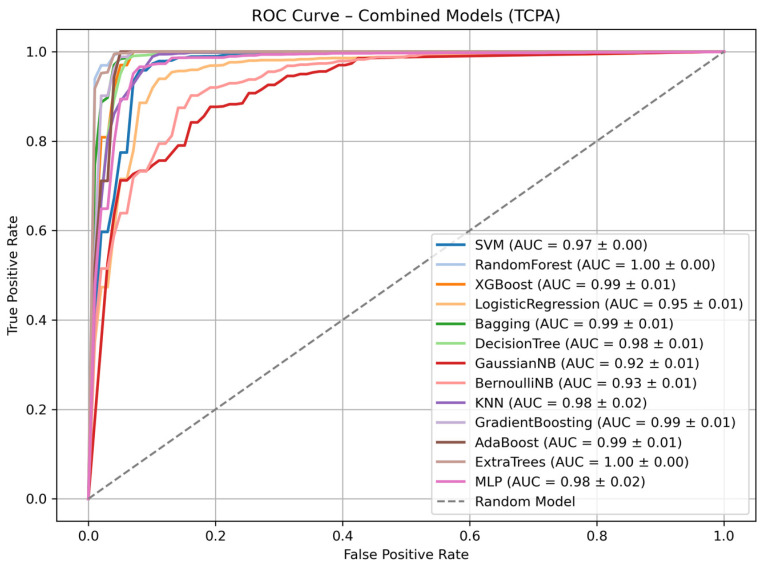
ROC curves for all thirteen supervised ML models applied to the TCPA dataset.

Figure 3 illustrates the ROC curves obtained from the stratified 5-fold cross-validation for the thirteen machine learning models evaluated on the TCPA dataset, highlighting their discriminative performance in distinguishing Responder from Non-Responder patients.

### 2.5. Comparative Analysis of Model Performance Across TCGA and TCPA Cohorts

**Figure 4:**
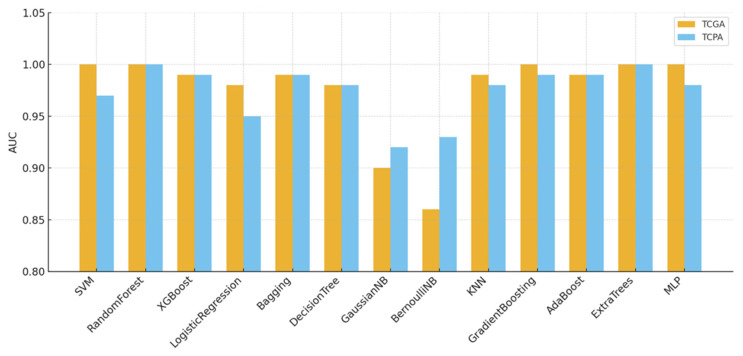
Comparison of ROC-AUC values obtained for the thirteen supervised machine learning models evaluated on the TCGA and TCPA cohorts. Bars represent the mean ROC-AUC values derived from stratified 5-fold cross-validation for each model. ROC-AUC values for the TCGA are shown in gold yellow, whereas ROC-AUC values for the TCPA cohort are shown in light blue.

To further assess the robustness and generalizability of the proposed ML framework, a comparative analysis of model performance across the TCGA and TCPA cohorts was conducted. Specifically, the ROC-AUC values obtained for each of the thirteen ML models were directly compared between the two independent datasets. Figure 4 presents a bar plot illustrating the mean ROC-AUC values achieved by each model on the TCGA and TCPA cohorts. Overall, the comparison reveals a high degree of consistency in model performance across datasets, with most algorithms maintaining similarly elevated AUC values in both cohorts. Ensemble-based models, including Random Forest, Gradient Boosting, AdaBoost, Bagging, and ExtraTrees, consistently achieved the highest discriminative performance, with ROC-AUC values approaching or exceeding 0.99 in both TCGA and TCPA datasets. Notably, ExtraTrees and Random Forest exhibited near-identical and exceptionally high ROC-AUC values across both cohorts, highlighting their strong robustness to dataset-specific variability and their ability to generalize across different proteomic sources. Similarly, Gradient Boosting and XGBoost demonstrated stable performance with minimal AUC fluctuations between TCGA and TCPA, reinforcing the reliability of boosting-based strategies in capturing biologically relevant patterns. Kernel-based and distance-based methods, such as Support Vector Machines and k-Nearest Neighbors, also showed comparable AUC values across datasets, albeit with slightly greater variability than ensemble approaches. In contrast, probabilistic classifiers, particularly Gaussian and Bernoulli Naive Bayes, consistently yielded lower ROC-AUC values and exhibited greater performance discrepancies between cohorts, underscoring their limited suitability for high-dimensional proteomic data. Collectively, this comparative analysis demonstrates that the proposed ML framework achieves highly consistent predictive performance across independent proteomic datasets, supporting its robustness and translational potential. The strong concordance of ROC-AUC values between TCGA and TCPA further validates the generalizability of ensemble-based models and justifies their selection for downstream biological interpretation and clinical decision-support applications.

#### Learning-Curve Analysis and Assessment of Overfitting

**Figure 5:**
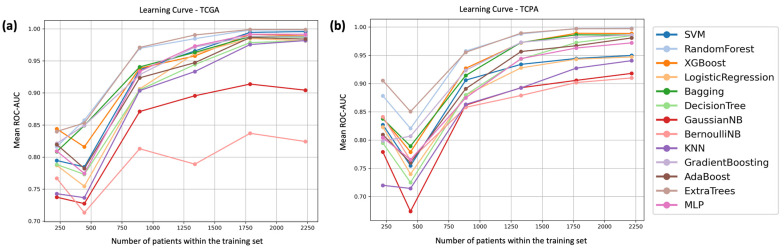
Learning curves showing ROC-AUC as a function of training set size for the thirteen supervised machine learning models evaluated on the TCGA (**a**) and TCPA (**b**) datasets. Performance stabilizes with increasing sample size, indicating robust generalization and limited overfitting.

To further assess model robustness and to mitigate the risk of overfitting, a learning-curve analysis was performed by evaluating model performance on progressively increasing subsets of the training data. Specifically, the ROC-AUC was computed for each classifier using training sets of increasing size, allowing direct assessment of how predictive performance evolved as a function of the number of available samples. As shown in Figure 5, all models exhibited a consistent improvement in ROC-AUC with increasing training set size on both the TCGA and TCPA cohorts, followed by a clear stabilization phase. This behavior indicates that model performance benefits from the inclusion of additional data and does not rely on memorization of a limited subset of samples. Importantly, no degradation in performance was observed as training size increased, suggesting that the models generalize well and are not affected by overfitting. Ensemble-based methods, including Random Forest, ExtraTrees, Gradient Boosting, and AdaBoost, consistently achieved higher ROC-AUC values across all training sizes and demonstrated faster convergence toward stable performance compared to other algorithms. Kernel-based and neural network models displayed a similar trend, although with slightly slower convergence. In contrast, simpler probabilistic classifiers showed lower and less stable performance, particularly for smaller training subsets. Overall, the learning-curve analysis confirms the robustness of the proposed machine learning framework and supports the validity of the reported performance results. The progressive stabilization of ROC-AUC values with increasing data size further demonstrates that the high predictive accuracy observed in both TCGA and TCPA analyses reflects genuine learning of biologically relevant patterns rather than overfitting to the training data (all the results have been obtained through Script S2 in Supplementary Material S3).

### 2.6. Feature Selection and Global SHAP-Based Ranking

To identify robust and model-independent biomarkers associated with therapeutic response, a two-step feature selection strategy was adopted and applied separately to the TCGA and TCPA datasets. In the first step, feature importance was computed independently for each of the thirteen supervised machine learning algorithms. For each model, the top 20 most informative proteomic features were identified based on model-specific importance measures. The complete set of algorithm-specific feature rankings is reported in the Supplementary Materials S2 and S3 (Script S3 and Figures S23–S48).

In the second step, a global consensus ranking was constructed for each dataset by aggregating the feature importance results obtained across all models. For each feature, a mean importance score was calculated by averaging normalized importance values across models, together with a support score representing the number of models in which the feature appeared among the top-ranked candidates. Features were subsequently ranked according to their mean importance, yielding a dataset-specific global ranking that highlights proteomic markers consistently selected across multiple learning paradigms. The global rankings reported in Table 3 summarize the most consistently selected features and do not reflect model-specific importance values. This consensus-based approach reduces dependence on individual model assumptions and enhances the robustness and interpretability of feature selection. The resulting global rankings were used to identify the most informative proteomic features for downstream biological interpretation and SHAP-based analysis (see Script S4 in Supplementary Material S3).

To further assess the robustness of the global consensus feature selection strategy, we performed an additional performance-weighted ranking analysis using Script S6 in Supplementary Material S3. In this analysis, model-specific normalized feature importance scores were weighted according to cross-validated ROC-AUC penalized by fold-to-fold variability, thereby assigning greater influence to features selected by more accurate and stable classifiers. The performance-weighted rankings were then compared with the original equal-weight consensus rankings reported in Table 3. This analysis showed very high agreement between the two approaches, with 29/30 shared features in the TCGA cohort and 30/30 shared features in the TCPA cohort, corresponding to Jaccard indices of 0.935 and 1.000, respectively (See Supplementary Material S2, Table S5 and Figures S49 and S50). These findings support the robustness of the selected proteomic signatures and indicate that the final biomarker panels are not primarily driven by averaging artifacts or by contributions from lower-performing classifiers.

The global consensus feature rankings identified several proteomic markers and signaling axes that are well established in breast cancer biology, supporting the biological plausibility of the proposed framework. Notably, a subset of proteins was consistently identified across both TCGA and TCPA datasets, indicating cross-dataset robustness of the consensus strategy. Shared top-ranked features included BCLXL, AMPKALPHA, RAPTOR, ANNEXIN1, IRS1, AKT_pS473, S6_pS235S236, SRC_pY416, CKIT, YAP_pS127, and CHK1_pS296, highlighting a core proteomic signature associated with therapeutic response in breast cancer.

Several of these shared proteins converge on the PI3K–AKT–mTOR signaling axis, including AKT_pS473, AMPKALPHA, RAPTOR, S6_pS235S236, and IRS1, as well as downstream translational regulators P70S6K1 and 4EBP1 identified in the TCGA ranking, underscoring the central role of metabolic control and growth signaling in breast cancer progression and treatment response [38,39,40,41,42,43]. Additional upstream and parallel modulators such as PDK1_pS241, PKCPANBETAII_pS660, ERK2, and GSK3_pS9 further reinforce the central role of interconnected kinase signaling cascades in regulating proliferation, metabolism, and therapeutic sensitivity in breast cancer. The coordinated selection of multiple nodes within these pathways across independent datasets suggests pathway-level dysregulation rather than isolated protein effects [44,45,46].

Proteins involved in cell survival and apoptosis regulation were also prominently represented. BCLXL, a key anti-apoptotic regulator, is widely implicated in chemoresistance and endocrine therapy resistance in breast cancer. Similarly, the identification of YAP_pS127, a component of the Hippo pathway, reinforces the importance of survival signaling and cellular plasticity mechanisms in therapeutic adaptation [47,48,49,50,51]. NOTCH1 signaling further contributes to this pro-survival and differentiation-related axis, which is frequently dysregulated in aggressive breast cancer subtypes [52].

Markers of DNA damage response and cell cycle control were consistently selected, including CHK1_pS296, KU80, MRE11, and PCNA, underscoring the tight link between genomic instability, replication stress, and therapeutic response. The presence of CYCLINB1 further reflects active regulation of cell cycle progression, a hallmark of proliferative breast tumors [53,54,55].

Metabolic reprogramming emerged as a dominant theme across the rankings. Proteins such as FASN, GAPDH, ERRalpha, TRAP1, DRP1, and AMPKALPHA point toward alterations in lipid biosynthesis, glycolysis, mitochondrial dynamics, oxidative phosphorylation, and energy sensing, all of which are increasingly recognized as critical determinants of treatment resistance and subtype-specific vulnerabilities in breast cancer [38,56,57,58,59,60]. The identification of PRDX1 further suggests involvement of redox homeostasis and oxidative stress adaptation mechanisms [61].

Transcriptional and epigenetic regulation were also prominently represented, with AR, CMYC, GATA6, XBP1, ENY2, ARID1A, and FTO implicating hormone signaling, transcriptional reprogramming, chromatin remodeling, and RNA modification processes. AR is particularly relevant in luminal and androgen receptor-positive triple-negative breast cancer subsets, while ARID1A alterations are linked to chromatin dysregulation and therapeutic response variability [62,63,64,65,66,67,68].

Signaling mediators involved in invasion, structural remodeling, and adaptive responses were consistently selected. SRC_pY416 and CKIT highlight activation of oncogenic kinase signaling, while SNAIL, MMP14, EGFR, and EphA2_pS897 support the involvement of epithelial–mesenchymal transition (EMT) programs and invasive behavior, processes strongly associated with aggressive and therapy-resistant breast cancer phenotypes [69,70,71]. Additional cytoskeletal regulators, including MYOSINIIA, MYH11, and TRANSGLUTAMINASE, further reinforce the contribution of cellular plasticity and motility. The recurrent identification of ANNEXIN1 suggests an additional role for inflammatory signaling and tumor–microenvironment interactions in modulating therapeutic response [72].

Autophagy and proteostasis-related mechanisms were implicated by Atg3 and P62LCKLIGAND, indicating potential roles for protein turnover and stress adaptation pathways in shaping treatment outcomes [73].

Beyond these shared biological themes, dataset-specific components further refined the regulatory landscape. In the TCGA cohort, proteins such as HSP70, LCK, KU80, and FASN emphasized stress response mechanisms, immune-related signaling, and DNA repair pathways [74]. Conversely, the TCPA dataset uniquely prioritized regulators including GAPDH, ARID1A, NOTCH1, PCNA, MRE11, BRAF, CMYC, CYCLINB1, and ERK2, reflecting additional layers of proliferative control, chromatin remodeling, MAPK pathway activation, and replication stress management [75].

Importantly, these dataset-specific features converge on biological processes already implicated by the shared core signature, suggesting that while a conserved backbone of survival, growth, metabolic adaptation, and genomic maintenance underlies therapeutic response in breast cancer, additional context-dependent regulators may emerge according to cohort composition, subtype distribution, and proteomic depth.

**Figure 6:**
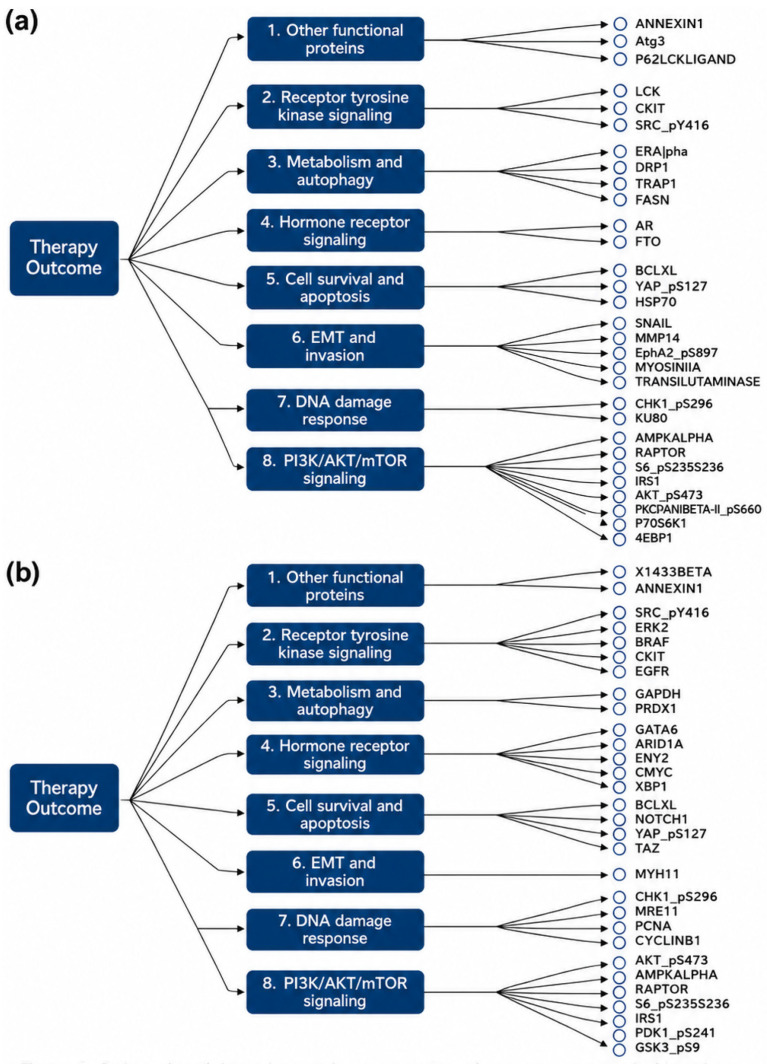
Pathway-based layered network representation of consensus proteomic biomarkers associated with therapy outcome in breast cancer for TCGA (**a**) and TCPA (**b**).

Collectively, the integrated representation of proliferative signaling, apoptosis resistance, metabolic rewiring, transcriptional plasticity, DNA repair, and invasive programs among the top-ranked features supports the biological coherence of the consensus-based machine learning framework. The convergence of these well-characterized breast cancer-related mechanisms reinforces both the robustness of the feature selection strategy and the translational relevance of the identified proteomic biomarkers. After identifying the convergence of top-ranked proteins within major signaling programs, we constructed a pathway-based layered network representation to visually integrate the functional relationships among the consensus biomarkers (Figure 6). This neural-style architecture highlights the hierarchical organization of therapy outcome-associated proteins into coordinated biological modules, reinforcing the pathway-level interpretation emerging from the feature ranking analysis.

Beyond predictive performance, we sought to determine whether the SHAP-derived proteomic signature reflected biologically coherent mechanisms associated with therapeutic response in breast cancer. To complement feature importance ranking with directional biological interpretation, we compared protein expression levels between Responders and Non-Responders within each cohort (TCGA and TCPA). For each of the 30 SHAP-selected proteins, group differences were assessed using the non-parametric Mann–Whitney U test, and multiple testing correction was performed using the Benjamini–Hochberg false discovery rate (FDR) procedure (Results are summarized in Table S4). The magnitude and direction of group differences were quantified using Cliff’s delta, a robust non-parametric effect size metric. Positive Cliff’s delta values indicate higher expression in Responders, whereas negative values indicate higher expression in Non-Responders.

In the TCGA cohort, multiple SHAP-prioritized proteins exhibited statistically significant and directionally consistent expression differences between Responders and Non-Responders. AKT_pS473, CHK1_pS296, and DRP1 showed significantly higher expression in Non-Responders, supporting enhanced survival signaling, replication stress adaptation, and metabolic reprogramming in resistant tumors [76,77,78]. Conversely, RAPTOR and additional mTOR-related components were relatively enriched in Responders, suggesting distinct pathway configurations potentially associated with therapeutic sensitivity.

A partially overlapping yet biologically coherent pattern was observed in the independent TCPA cohort. Proteins including MRE11 and RAPTOR were preferentially expressed in Responders, whereas AKT_pS473 and ANNEXIN1 were enriched in Non-Responders [76,77,79], reinforcing the reproducibility of directional associations across datasets. Importantly, several proteins ranking highly in SHAP importance also demonstrated significant Responder/Non-Responder expression differences, providing orthogonal validation of the machine learning-derived signature.

**Figure 7:**
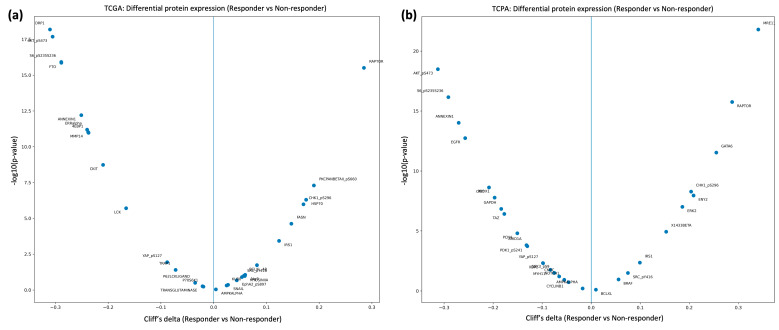
Volcano plots showing differences in protein expression between Responders and Non-Responders for the 30 SHAP-selected proteins in TCGA (**a**) and TCPA (**b**) cohorts. The *x*-axis represents the direction and magnitude of the effect (positive values indicate higher expression in Responders; negative values indicate higher expression in Non-Responders), while the *y*-axis represents statistical significance.

Collectively, these results indicate that the predictive framework captures biologically meaningful proteomic alterations linked to therapeutic response and resistance mechanisms, rather than merely statistical artifacts. The convergence of survival signaling, DNA damage response activation, metabolic adaptation, and kinase pathway dysregulation across independent datasets further strengthens the translational relevance of the identified biomarker panel. Volcano plots summarizing effect size versus statistical significance are presented in Figure 7 (see Script S5 in Supplementary Material S3).

#### Impact of Feature Reduction on Model Performance

**Figure 8:**
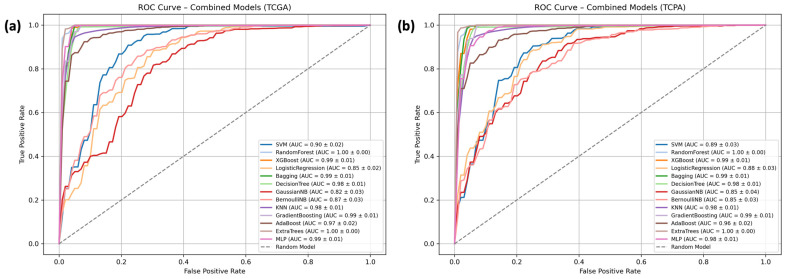
ROC curves obtained from stratified 5-fold cross-validation for the thirteen supervised machine learning models evaluated using the reduced feature set consisting of the top 30 globally ranked proteomic features. (**a**) Performance on the TCGA breast cancer cohort; (**b**) Performance on the TCPA breast cancer cohort.

The final evaluation assessed whether a compact and interpretable proteomic signature could retain the strong predictive performance observed throughout the study. As shown in Figure 8, re-computing model performance using only the top 30 globally ranked features resulted in minimal performance degradation across all evaluated scenarios. On the Breast cancer cohorts (Figure 8a,b), ensemble-based classifiers consistently achieved near-perfect ROC-AUC values, demonstrating that the majority of predictive information is captured by a limited subset of features rather than by high-dimensional proteomic profiles. Collectively, these results provide strong evidence that the proposed framework identifies a stable, biologically meaningful, and parsimonious set of proteomic features capable of supporting accurate therapeutic response prediction across independent datasets and clinically relevant subgroups. This finding underscores the translational potential of the approach, as it demonstrates that high predictive performance can be achieved using a limited and interpretable molecular signature rather than relying on extensive high-dimensional measurements.

## 3. Materials and Methods

### 3.1. Data Collection and Cohort Definition

Proteomic, clinical, and therapeutic data were collected from publicly available cancer genomics and proteomics repositories. Proteomic expression profiles were obtained from The Cancer Genome Atlas (TCGA, https://portal.gdc.cancer.gov/; accessed on 31 May 2026) [22] and The Cancer Proteome Atlas (TCPA, https://tcpa.drbioright.org/rppa500/main.html; accessed on 31 May 2026) [23,24,25], while clinical annotations, survival outcomes, and pharmacological treatment information were retrieved from cBioPortal (https://www.cbioportal.org/; accessed on 31 May 2026) [26,27,28]. Only patients diagnosed with breast cancer were considered for this study. Clinical data extracted from cBioPortal included tumor characteristics (American Joint Committee on Cancer stage, cancer type, and molecular subtype), clinical outcomes (new neoplasm events and disease-specific survival status), and pharmacological therapies (drug name and therapy type). Proteomic data, consisting of protein expression profiles measured by RPPA technology, were retrieved separately from TCGA and TCPA repositories. Initially, proteomic data originating from TCGA and TCPA were maintained as independent datasets to preserve data provenance and allow subsequent harmonization steps.

Patient- and sample-level identifiers were used to accurately match proteomic profiles with the corresponding clinical and therapeutic information. Only samples for which complete and consistent multidimensional data were available were retained for downstream analyses. Patients lacking either proteomic measurements or pharmacological treatment annotations were excluded. Following data integration, two analytical cohorts were defined (TCGA and TCPA). The Breast Cancer cohort included all breast cancer patients meeting the inclusion criteria.

### 3.2. Data Preprocessing and Integration

Following data collection, proteomic, clinical, and therapeutic datasets were subjected to preprocessing and integration procedures to ensure consistency and suitability for machine learning analyses. Proteomic expression matrices obtained from TCGA and TCPA were processed independently to preserve dataset-specific characteristics and enable cross-cohort validation.

Missing values were systematically assessed across proteomic features. Proteins exhibiting excessive missingness across samples were excluded from downstream analyses to reduce noise and improve model robustness. Only features with sufficient data coverage were retained. Clinical and therapeutic data were filtered to remove incomplete or inconsistent records.

Proteomic and clinical datasets were then integrated using unique patient and sample identifiers, ensuring accurate correspondence between molecular profiles, treatment information, and clinical outcomes. Only patients with complete proteomic profiles and associated therapeutic response annotations were retained in the final integrated datasets used for model development.

Therapy-response labels were assigned by integrating treatment-response annotations with clinical outcome information. When available, treatment_best_response was used as the primary response-related variable; however, because this field was not uniformly available across all patients, disease-specific survival status, overall survival status, disease-free status, and new neoplasm events after initial therapy were also considered. Patients reported as alive or deceased but tumor-free were classified as Responders, whereas patients with persistent disease, recurrence or progression, new neoplasm events after initial therapy, or tumor-related death were classified as Non-Responders.

Because treatment annotations in public repositories were heterogeneous and not uniformly available at the regimen level, therapy-response modeling was performed across the overall treated cohort. Drug names were included as one-hot encoded variables to retain treatment-related information within the feature space. Accordingly, the model was designed to predict an overall therapy-response phenotype in a treatment-informed context, rather than response to a single drug or specific therapeutic regimen.

### 3.3. Machine Learning Models

A total of thirteen supervised machine learning algorithms were evaluated in this study to model the relationship between proteomic profiles and therapeutic response. The selected classifiers represent a broad range of learning paradigms and included: Support Vector Machine (SVM), Random Forest, XGBoost, Logistic Regression, Bagging, Decision Tree, Gaussian Naive Bayes, Bernoulli Naive Bayes, k-Nearest Neighbors (kNN), Gradient Boosting, AdaBoost, ExtraTrees, and Multilayer Perceptron (MLP).

These algorithms were selected based on their established effectiveness in high-dimensional biomedical datasets and their complementary strengths in modeling linear and non-linear relationships. All computational analyses were performed in Python v3.9.19 using scikit-learn v1.6.1, XGBoost v2.1.4, SHAP v0.47.2, NumPy v2.0.2, pandas v2.2.2, Matplotlib v3.9.2, and SciPy v1.13.1. The complete scripts used for model training, evaluation, feature selection, SHAP-based interpretation, and statistical analysis are provided in Supplementary Material S3. Unless otherwise specified, default parameters were used as initialization prior to hyperparameter optimization.

### 3.4. Model Training and Evaluation Strategy

Model training and evaluation were performed using a stratified 5-fold cross-validation strategy to ensure balanced representation of Responder and Non-Responder classes in each fold. In each iteration, four folds were used for model training, while the remaining fold was reserved for validation. This procedure was repeated five times, allowing each fold to serve once as the validation set.

Hyperparameter optimization was conducted within the cross-validation framework using a Grid-search strategy. For each algorithm, predefined combinations of hyperparameters were systematically explored, and the configuration yielding the best cross-validated ROC-AUC was selected as the optimal model setting.

Model performance was evaluated using multiple classification metrics, including accuracy, precision, recall, F1-score, and the Area Under the Receiver Operating Characteristic Curve (ROC-AUC). ROC-AUC was used as the primary metric to assess discriminative performance across varying classification thresholds.

All reported performance metrics represent the mean and standard deviation across the five cross-validation folds. ROC-AUC values obtained using the optimal hyperparameter configuration for each algorithm are summarized as heatmaps in the Supplementary Material S2 and in Table S3.

### 3.5. Feature Selection Strategy

To identify robust and model-independent proteomic biomarkers associated with therapeutic response, a two-step feature selection strategy was adopted. In the first step, feature importance was computed independently for each machine learning algorithm using model-specific importance measures. For each classifier, the top-ranked proteomic features were identified.

In the second step, a global consensus ranking was constructed for each dataset by aggregating feature importance scores across all algorithms. Features were ranked based on their average normalized importance and consistency of selection across models. This approach reduced dependence on individual model assumptions and highlighted proteomic features consistently associated with therapeutic response.

Based on the global ranking, a reduced feature set consisting of the top 30 proteomic features was selected for downstream analyses. To evaluate the impact of dimensionality reduction on predictive performance, machine learning models were retrained using only the reduced set of 30 selected proteomic features. The same algorithms, hyperparameter configurations, and stratified cross-validation strategy described above were retained to ensure fair comparison with models trained on the full feature space.

Model interpretability was assessed using SHapley Additive exPlanations (SHAP) to quantify the contribution of individual proteomic features to model predictions [37]. SHAP values were computed for selected models to identify features with the greatest impact on therapeutic response classification.

To assess whether the SHAP-selected proteomic features exhibited directional expression differences between clinical response groups, differential expression analysis was performed independently within each cohort (TCGA and TCPA).

For each of the 30 SHAP-selected proteins, expression levels were compared between Responders and Non-Responders using the non-parametric Mann–Whitney U test. This test was selected due to its robustness to non-normal data distributions and unequal variances between groups [80].

To control for multiple hypothesis testing across the 30 proteins, *p*-values were adjusted using the Benjamini–Hochberg false discovery rate (FDR) correction procedure. Adjusted *p*-values (FDR) were used to determine statistical significance [81].

In addition to statistical significance, the magnitude and direction of group differences were quantified using Cliff’s delta, a non-parametric effect size metric ranging from −1 to +1. Positive Cliff’s delta values indicate higher protein expression in Responders, whereas negative values indicate higher expression in Non-Responders [82].

Volcano plots were generated to visualize the relationship between effect size (Cliff’s delta) and statistical significance (−log10 *p*-value) for each protein. All analyses were performed separately for the TCGA and TCPA cohorts.

## 4. Conclusions

In this study, we developed and validated B.R.E.A.S.T. (Breast canceR Enhanced AI-Supported Therapy), an interpretable machine learning framework designed to predict therapeutic response in breast cancer by integrating tumor proteomic profiles with clinical and treatment-related data. Through systematic benchmarking of thirteen supervised learning algorithms across two independent large-scale datasets (TCGA and TCPA), the framework demonstrated stable and reproducible predictive performance, supporting the feasibility of proteomics-driven response modeling in heterogeneous breast cancer populations [83].

A central strength of this work lies in the rigorous evaluation strategy adopted throughout model development. The use of stratified cross-validation, learning-curve analyses, and cross-cohort comparisons allowed us to assess not only predictive accuracy but also model robustness and generalizability. The consistent performance observed across independent datasets suggests that the predictive capacity of the framework is driven by biologically meaningful molecular patterns rather than cohort-specific biases or overfitting phenomena.

Beyond predictive performance, this study emphasizes interpretability as a core component of translational machine learning. The two-step feature selection strategy enabled the identification of a compact yet biologically coherent proteomic signature. Remarkably, retraining models using only the top 30 globally ranked proteins preserved high predictive accuracy, underscoring the potential of parsimonious biomarker panels to capture key determinants of therapeutic response while enhancing model transparency and clinical feasibility.

Importantly, the integration of SHAP-based interpretability and subsequent directional expression analyses revealed convergence between model-derived importance and established oncogenic pathways. Proteins implicated in survival signaling, DNA damage response, metabolic reprogramming, and invasion-associated processes emerged as recurrent contributors to response stratification across both cohorts. The consistency between SHAP prioritization and Responder/Non-Responder-associated expression differences further strengthens the biological plausibility and mechanistic coherence of the identified signature.

From a translational perspective, these findings suggest that large-scale proteomic profiling, when coupled with interpretable machine learning, can move beyond purely predictive modeling toward mechanistically informed treatment stratification. While prospective validation in independent clinical cohorts remains essential prior to clinical implementation, the present framework establishes a methodological blueprint for integrating proteomics, algorithmic benchmarking, and interpretability-driven biomarker selection within a precision oncology context.

The Responder/Non-Responder classification used in this study was based on clinically meaningful treatment response and outcome annotations available from public repositories, including best treatment response, survival status, disease-free status, and post-treatment progression information. However, these retrospective annotations do not correspond to a uniformly standardized RECIST-based endpoint across all patients. Future prospective validation using harmonized and regimen-specific response criteria will further strengthen the clinical applicability of the proposed framework.

Another important limitation concerns treatment specificity. In the present study, B.R.E.A.S.T. was designed to predict an overall therapy-response phenotype rather than response to a single drug or specific therapeutic regimen. Although drug names were one-hot encoded to retain treatment-related information within the model, patients received heterogeneous therapies with distinct mechanisms of action, including chemotherapy, endocrine therapy, targeted therapy, and combined regimens. Therefore, aggregation across treatment types may partially conflate therapy-specific biological mechanisms. Future studies should include sensitivity analyses by treatment category when sufficiently powered, balanced, and uniformly annotated cohorts become available, as unbalanced subgroup sizes could otherwise bias therapy-specific analyses.

Collectively, B.R.E.A.S.T. provides a robust, modular, and extensible platform for therapy response prediction in breast cancer. By combining predictive strength with biological insight, the framework offers a scalable foundation for future prospective studies and supports the broader integration of proteomics-informed artificial intelligence into precision medicine strategies.

## Figures and Tables

**Table 1:** Performance comparison of thirteen supervised machine learning classifiers on the TCGA dataset. Metrics are reported as mean ± standard deviation over stratified 5-fold cross-validation.

Model	Accuracy	Precision	Recall	F1 Score	ROC AUC
SVM	0.984 ± 0.004	0.983 ± 0.005	0.999 ± 0.001	0.991 ± 0.003	0.997 ± 0.002
RandomForest	0.996 ± 0.004	0.995 ± 0.004	1.000 ± 0.000	0.998 ± 0.002	0.997 ± 0.003
XGBoost	0.989 ± 0.007	0.987 ± 0.008	1.000 ± 0.000	0.994 ± 0.004	0.993 ± 0.006
LogisticRegression	0.987 ± 0.004	0.995 ± 0.005	0.990 ± 0.004	0.992 ± 0.002	0.982 ± 0.014
Bagging	0.996 ± 0.004	0.995 ± 0.004	1.000 ± 0.000	0.998 ± 0.002	0.992 ± 0.009
DecisionTree	0.988 ± 0.006	0.995 ± 0.004	0.991 ± 0.005	0.993 ± 0.004	0.981 ± 0.013
GaussianNB	0.589 ± 0.020	0.987 ± 0.003	0.527 ± 0.022	0.687 ± 0.019	0.904 ± 0.008
BernoulliNB	0.845 ± 0.011	0.926 ± 0.012	0.891 ± 0.011	0.908 ± 0.007	0.858 ± 0.018
KNN	0.993 ± 0.004	0.995 ± 0.004	0.996 ± 0.002	0.996 ± 0.002	0.989 ± 0.011
GradientBoosting	0.996 ± 0.003	0.995 ± 0.004	0.999 ± 0.001	0.997 ± 0.002	0.996 ± 0.003
AdaBoost	0.996 ± 0.003	0.995 ± 0.004	0.999 ± 0.001	0.997 ± 0.002	0.989 ± 0.010
ExtraTrees	0.996 ± 0.004	0.995 ± 0.004	1.000 ± 0.000	0.998 ± 0.002	0.999 ± 0.001
MLP	0.984 ± 0.006	0.994 ± 0.005	0.988 ± 0.006	0.991 ± 0.004	0.995 ± 0.004

**Table 2:** Performance comparison of thirteen supervised machine learning classifiers on the TCPA dataset. Metrics are reported as mean ± standard deviation over stratified 5-fold cross-validation.

Model	Accuracy	Precision	Recall	F1 Score	ROC AUC
SVM	0.969 ± 0.005	0.978 ± 0.003	0.987 ± 0.005	0.982 ± 0.003	0.970 ± 0.003
RandomForest	0.996 ± 0.004	0.995 ± 0.004	1.000 ± 0.000	0.998 ± 0.002	0.998 ± 0.002
XGBoost	0.991 ± 0.004	0.990 ± 0.004	1.000 ± 0.000	0.995 ± 0.002	0.988 ± 0.010
LogisticRegression	0.935 ± 0.017	0.978 ± 0.008	0.945 ± 0.016	0.961 ± 0.010	0.953 ± 0.015
Bagging	0.996 ± 0.004	0.995 ± 0.004	1.000 ± 0.000	0.998 ± 0.002	0.993 ± 0.006
DecisionTree	0.982 ± 0.008	0.993 ± 0.006	0.986 ± 0.006	0.989 ± 0.005	0.982 ± 0.011
GaussianNB	0.724 ± 0.026	0.990 ± 0.002	0.685 ± 0.030	0.809 ± 0.022	0.918 ± 0.006
BernoulliNB	0.924 ± 0.009	0.939 ± 0.009	0.975 ± 0.005	0.957 ± 0.005	0.935 ± 0.012
KNN	0.979 ± 0.007	0.987 ± 0.005	0.989 ± 0.005	0.988 ± 0.004	0.979 ± 0.017
GradientBoosting	0.996 ± 0.004	0.995 ± 0.004	1.000 ± 0.000	0.998 ± 0.002	0.991 ± 0.008
AdaBoost	0.995 ± 0.005	0.995 ± 0.004	0.999 ± 0.002	0.997 ± 0.003	0.987 ± 0.011
ExtraTrees	0.995 ± 0.004	0.995 ± 0.004	0.999 ± 0.001	0.997 ± 0.002	0.998 ± 0.002
MLP	0.969 ± 0.008	0.984 ± 0.004	0.980 ± 0.008	0.982 ± 0.005	0.975 ± 0.017

**Table 3:** Global consensus ranking of proteomic features for the TCGA and TCPA dataset. Features were ranked according to their mean normalized importance across thirteen supervised machine learning models. The “Models Supporting” column indicates the number of classifiers in which each feature appeared among the top 30 ranked candidates, providing a measure of cross-model robustness. Standard deviation reflects variability in feature importance across models.

TCGA			TCPA		
Feature	Mean Importance	Std Importance	Feature	Mean Importance	Std Importance
CHK1_pS296	0.440	0.415	GAPDH	0.476	0.410
BCLXL	0.400	0.369	BCLXL	0.455	0.366
HSP70	0.371	0.389	X1433BETA	0.432	0.415
SNAIL	0.284	0.260	ARID1A	0.356	0.297
LCK	0.281	0.353	SRC_pY416	0.350	0.330
AMPKALPHA	0.271	0.338	AKT_pS473	0.318	0.253
MMP14	0.265	0.310	NOTCH1	0.264	0.300
EphA2_pS897	0.246	0.197	AMPKALPHA	0.233	0.278
ANNEXIN1	0.241	0.291	ERK2	0.222	0.263
RAPTOR	0.238	0.262	RAPTOR	0.209	0.308
CKIT	0.211	0.229	CYCLINB1	0.207	0.166
S6_pS235S236	0.190	0.164	XBP1	0.207	0.194
DRP1	0.184	0.149	PDK1_pS241	0.203	0.287
KU80	0.183	0.206	MYH11	0.198	0.190
IRS1	0.181	0.239	PRDX1	0.196	0.264
FTO	0.180	0.294	ANNEXIN1	0.194	0.226
ERRalpha	0.180	0.208	GATA6	0.190	0.243
YAP_pS127	0.175	0.236	ENY2	0.188	0.139
AKT_pS473	0.164	0.127	S6_pS235S236	0.186	0.181
P62LCKLIGAND	0.161	0.163	EGFR	0.181	0.264
TRAP1	0.161	0.246	TAZ	0.166	0.250
TRANSGLUTAMINASE	0.160	0.152	BRAF	0.166	0.258
FASN	0.155	0.192	IRS1	0.164	0.132
PKCPANBETAII_pS660	0.153	0.5	CMYC	0.163	0.125
P70S6K1	0.153	0.182	PCNA	0.161	0.197
Atg3	0.150	0.250	CKIT	0.159	0.273
SRC_pY416	0.149	0.242	MRE11	0.154	0.162
4EBP1	0.149	0.184	GSK3_pS9	0.153	0.122
AR	0.149	0.166	YAP_pS127	0.152	0.206
MYOSINIIA	0.148	0.208	CHK1_pS296	0.151	0.193

## Data Availability

The datasets analyzed in this study were obtained from publicly available repositories, including The Cancer Genome Atlas (TCGA), The Cancer Proteome Atlas (TCPA), and cBioPortal, as described in Section 3. The processed data matrices, supplementary tables, figures, and scripts supporting the analyses are provided in the Supplementary Materials.

## Supplementary Materials

The following supporting information can be downloaded at: https://www.mdpi.com/article/10.3390/ijms27125163/s1.

## Abbreviations

The following abbreviations are used in this manuscript:

AI	Artificial Intelligence
AJCC	American Joint Committee on Cancer
AMPK	AMP-activated protein kinase
AMPKALPHA	AMP-activated protein kinase alpha
ANNEXIN1	Annexin A1
AR	Androgen Receptor
ARID1A	AT-rich interaction domain-containing protein 1A
AUC	Area Under the Curve
Atg3	Autophagy-related protein 3
BC	Breast Cancer
BCLXL	B-cell lymphoma-extra large
BNB	Bernoulli Naive Bayes
BRAF	B-Raf proto-oncogene
B.R.E.A.S.T.	Breast canceR Enhanced AI-Supported Therapy
cBioPortal	Cancer Bioinformatics Portal
CHK1	Checkpoint Kinase 1
CHK1_pS296	Checkpoint Kinase 1 phosphorylated at serine 296
CKIT/c-KIT	KIT proto-oncogene receptor tyrosine kinase
Cliff’s delta	Non-parametric effect size measure
CMYC/c-MYC	MYC proto-oncogene
CYCLINB1	Cyclin B1
DNA	Deoxyribonucleic Acid
DRP1	Dynamin-Related Protein 1
DT	Decision Tree
EGFR	Epidermal Growth Factor Receptor
EMT	Epithelial–Mesenchymal Transition
ENY2	ENY2 transcription and mRNA export factor
EphA2_pS897	EphA2 phosphorylated at serine 897
ERK2	Extracellular Signal-Regulated Kinase 2
ERRalpha/ERRα	Estrogen-Related Receptor alpha
ExtraTrees	Extremely Randomized Trees
F1-score	Harmonic mean of precision and recall
FASN	Fatty Acid Synthase
FDR	False Discovery Rate
FPR	False Positive Rate
FTO	Fat mass and obesity-associated protein
GAPDH	Glyceraldehyde-3-Phosphate Dehydrogenase
GATA6	GATA binding protein 6
GB	Gradient Boosting
GNB	Gaussian Naive Bayes
GSK3_pS9	Glycogen synthase kinase 3 phosphorylated at serine 9
HSP70	Heat Shock Protein 70
IRS1	Insulin Receptor Substrate 1
kNN/KNN	k-Nearest Neighbors
KU80	Ku80 DNA repair protein/XRCC5
LCK	Lymphocyte-specific protein tyrosine kinase
LGR	Logistic Regression
MAPK	Mitogen-Activated Protein Kinase
ML	Machine Learning
MLP	Multilayer Perceptron
MMP14	Matrix Metalloproteinase 14
MRE11	MRE11 homolog, double-strand break repair nuclease
mTOR	mechanistic Target Of Rapamycin
MYH11	Myosin Heavy Chain 11
MYOSINIIA	Myosin IIA
NOTCH1	Notch Receptor 1
PCNA	Proliferating Cell Nuclear Antigen
PDK1	3-Phosphoinositide-Dependent Protein Kinase 1
PDK1_pS241	PDK1 phosphorylated at serine 241
PI3K	Phosphoinositide 3-Kinase
PKCPANBETAII_pS660	Protein Kinase C pan-beta II phosphorylated at serine 660
PRDX1	Peroxiredoxin 1
P62LCKLIGAND	p62 Lck ligand
P70S6K1	Ribosomal Protein S6 Kinase Beta-1
RAPTOR	Regulatory Associated Protein of mTOR
RECIST	Response Evaluation Criteria in Solid Tumors
RF	Random Forest
RNA	Ribonucleic Acid
ROC	Receiver Operating Characteristic
ROC-AUC	Area Under the Receiver Operating Characteristic Curve
RPPA	Reverse Phase Protein Array
S6	Ribosomal Protein S6
S6_pS235S236	Ribosomal Protein S6 phosphorylated at serines 235/236
SHAP	SHapley Additive exPlanations
SNAIL	Snail family transcription factor
SRC	SRC proto-oncogene, non-receptor tyrosine kinase
SRC_pY416	SRC phosphorylated at tyrosine 416
Std	Standard deviation
SVM	Support Vector Machine
TAZ	Transcriptional co-Activator with PDZ-binding motif
TCGA	The Cancer Genome Atlas
TCPA	The Cancer Proteome Atlas
TPR	True Positive Rate
TRAP1	TNF Receptor-Associated Protein 1
TRANSGLUTAMINASE	Transglutaminase
X1433BETA	14-3-3 beta protein
XBP1	X-box Binding Protein 1
XGBoost	eXtreme Gradient Boosting
YAP	Yes-Associated Protein
YAP_pS127	YAP phosphorylated at serine 127
z-score	Standardized score
