# Supplementary material for: B.R.E.A.S.T. Breast canceR Enhanced AI-Supported Therapy: A New Interpretable Proteomics-Driven Machine Learning Framework for Therapy Response Prediction in Breast Cancer
Source: Int J Mol Sci. 2026 Jun 6;27(12):5163. doi: 10.3390/ijms27125163 (PMC13299364; doi:10.3390/ijms27125163)
Supplement: Supplementary file 1 [file ijms-27-05163-s001.zip › Supplementary Material/Supplementary Material S2.pdf]

Supporting Material

B.R.E.A.S.T. Breast canceR Enhanced AI-Supported Therapy: A New Interpretable Proteomics-Driven Machine Learning Framework for Therapy Response Prediction in Breast Cancer

Alessia Bono [1,2], Gabriele La Monica [1], Federica Alamia [1], Dennis Tocco [1], Antonino Lauria [1,*] and Annamaria Martorana [1]

[1] Dipartimento di Scienze e Tecnologie Biologiche Chimiche e Farmaceutiche "STEBICEF", University of Palermo, Viale delle Scienze, Ed. 17, 90128 Palermo, Italy; alessia.bono01@unipa.it (A.B.); gabriele.lamonica01@unipa.it (G.L.M.); federica.alamia01@unipa.it (F.A.); dennis.tocco@unipa.it (D.T.); annamaria.martorana@unipa.it (A.M.)

[2] Fondazione Umberto Veronesi (FUV), via Solferino 19, 20121 Milano, Italy

* Correspondence: antonino.lauria@unipa.it; Tel.: +39-091238-96818

Table of contents

Supporting material:

- **Table S1:** Integrated data matrix for the TCGA breast cancer cohort (attached .xlsx file).
- **Table S2:** Integrated data matrix for the TCPA breast cancer cohort (attached .xlsx file).
- **Figures S1–22:** Heatmaps of ROC-AUC values obtained using the optimal hyperparameter configuration for each of the thirteen classifiers applied on the TCGA/TCPA dataset.
    - *S1*: Heatmap of ROC-AUC values obtained using the optimal hyperparameter configuration for the Support Vector Machine classifier applied on the TCGA dataset.
    - *S2*: Heatmap of ROC-AUC values obtained using the optimal hyperparameter configuration for the Random Forest classifier applied on the TCGA dataset.
    - *S3*: Heatmap of ROC-AUC values obtained using the optimal hyperparameter configuration for the XGBoost classifier applied on the TCGA dataset.
    - *S4*: Heatmap of ROC-AUC values obtained using the optimal hyperparameter configuration for the Logistic Regression classifier applied on the TCGA dataset.
    - *S5:* Heatmap of ROC-AUC values obtained using the optimal hyperparameter configuration for the Bagging classifier applied on the TCGA dataset.
    - *S6*: Heatmap of ROC-AUC values obtained using the optimal hyperparameter configuration for the DecisionTree classifier applied on the TCGA dataset.
    - *S7*: Heatmap of ROC-AUC values obtained using the optimal hyperparameter configuration for the k-Nearest Neighbors classifier applied on the TCGA dataset.
    - *S8*: Heatmap of ROC-AUC values obtained using the optimal hyperparameter configuration for the Gradient Boosting classifier applied on the TCGA dataset.
    - *S9*: Heatmap of ROC-AUC values obtained using the optimal hyperparameter configuration for the AdaBoost classifier applied on the TCGA dataset.
    - *S10*: Heatmap of ROC-AUC values obtained using the optimal hyperparameter configuration for the ExtraTrees classifier applied on the TCGA dataset.

- *S11:* Heatmap of ROC-AUC values obtained using the optimal hyperparameter configuration for the Multilayer Perceptron classifier applied on the TCGA dataset.
- *S12*: Heatmap of ROC-AUC values obtained using the optimal hyperparameter configuration for the Support Vector Machine classifier applied on the TCPA dataset.
- *S13:* Heatmap of ROC-AUC values obtained using the optimal hyperparameter configuration for the Random Forest classifier applied on the TCPA dataset.
- *S14*: Heatmap of ROC-AUC values obtained using the optimal hyperparameter configuration for the XGBoost classifier applied on the TCPA dataset.
- *S15*: Heatmap of ROC-AUC values obtained using the optimal hyperparameter configuration for the Logistic Regression classifier applied on the TCPA dataset.
- *S16*: Heatmap of ROC-AUC values obtained using the optimal hyperparameter configuration for the Bagging classifier applied on the TCPA dataset.
- *S17*: Heatmap of ROC-AUC values obtained using the optimal hyperparameter configuration for the DecisionTree classifier applied on the TCPA dataset.
- *S18*: Heatmap of ROC-AUC values obtained using the optimal hyperparameter configuration for the k-Nearest Neighbors classifier applied on the TCPA dataset.
- *S19:* Heatmap of ROC-AUC values obtained using the optimal hyperparameter configuration for the Gradient Boosting classifier applied on the TCPA dataset.
- *S20*: Heatmap of ROC-AUC values obtained using the optimal hyperparameter configuration for the AdaBoost classifier applied on the TCPA dataset.
- *S21*: Heatmap of ROC-AUC values obtained using the optimal hyperparameter configuration for the ExtraTrees classifier applied on the TCPA dataset.
- *S22*: Heatmap of ROC-AUC values obtained using the optimal hyperparameter configuration for the Multilayer Perceptron classifier applied on the TCPA dataset.

- **Table S3:** Hyperparameter search space and optimal configurations identified for each machine learning algorithm.
- **Figures S23–48:** Feature importance profiles obtained for each of the thirteen supervised machine learning algorithms applied on the TCGA/TCPA dataset.
    - *S23*: Feature importance analysis for the Support Vector Machine classifier applied to the TCGA dataset.
    - *S24*: Feature importance analysis for the Random Forest classifier applied to the TCGA dataset.
    - *S25*: Feature importance analysis for the XGBoost classifier applied to the TCGA dataset.
    - *S26*: Feature importance analysis for the Logistic Regression classifier applied to the TCGA dataset.
    - *S27:* Feature importance analysis for the Bagging classifier applied to the TCGA dataset.
    - *S28*: Feature importance analysis for the DecisionTree classifier applied to the TCGA dataset.
    - *S29*: Feature importance analysis for the GaussianNB classifier applied to the TCGA dataset.
    - *S30*: Feature importance analysis for the BernoulliNB classifier applied to the TCGA dataset.
    - *S31*: Feature importance analysis for the k-Nearest Neighbors classifier applied to the TCGA dataset.
    - *S32*: Feature importance analysis for the Gradient Boosting classifier applied to the TCGA dataset.
    - *S33:* Feature importance analysis for the AdaBoost classifier applied to the TCGA dataset.
    - *S34*: Feature importance analysis for the ExtraTrees classifier applied to the TCGA dataset.
    - *S35:* Feature importance analysis for the Multilayer Perceptron classifier applied to the TCGA dataset.

- *S36*: Feature importance analysis for the Support Vector Machine classifier applied to the TCPA dataset.
- *S37*: Feature importance analysis for the Random Forest classifier applied to the TCPA dataset.
- *S38*: Feature importance analysis for the XGBoost classifier applied to the TCPA dataset.
- *S39*: Feature importance analysis for the Logistic Regression classifier applied to the TCPA dataset.
- *S40*: Feature importance analysis for the Bagging classifier applied to the TCPA dataset.
- *S41:* Feature importance analysis for the DecisionTree classifier applied to the TCPA dataset.
- *S42*: Feature importance analysis for the GaussianNB classifier applied to the TCPA dataset.
- *S43*: Feature importance analysis for the BernoulliNB classifier applied to the TCPA dataset.
- *S44*: Feature importance analysis for the k-Nearest Neighbors classifier applied to the TCPA dataset.
- *S45*: Feature importance analysis for the Gradient Boosting classifier applied to the TCPA dataset.
- *S46*: Feature importance analysis for the AdaBoost classifier applied to the TCPA dataset.
- *S47*: Feature importance analysis for the ExtraTrees classifier applied to the TCPA dataset.
- *S48*: Feature importance analysis for the Multilayer Perceptron classifier applied to the TCPA dataset.

- **Table S4:** Differential expression analysis of SHAP-selected proteomic features in TCGA and TCPA cohorts.
- **Table S5:** Robustness analysis comparing equal-weight and performance-weighted consensus feature rankings.
- **Figure S49:** Performance-weighted consensus ranking of the top 30 proteomic features for the TCGA cohort.
- **Figure S50:** Performance-weighted consensus ranking of the top 30 proteomic features for the TCPA cohort.

Figures S1-22 provide additional details on model optimization and performance evaluation supporting the results presented in the main manuscript. For each of the thirteen supervised machine learning algorithms, hyperparameter tuning was performed to identify the optimal configuration maximizing predictive performance. Model optimization was conducted within the cross-validation framework described in the Methods section.

Using the optimal hyperparameter combination identified for each algorithm, the Area Under the Receiver Operating Characteristic Curve (ROC-AUC) was computed to quantify discriminative performance under optimized conditions. These ROC-AUC values reflect the maximum classification performance achievable by each model after hyperparameter selection.
To facilitate comparative analysis across algorithms and datasets, ROC-AUC values obtained with optimized hyperparameters are summarized using heatmap visualizations. Separate heatmaps are reported for the TCGA and TCPA datasets, enabling direct comparison of model performance patterns and consistency across independent proteomic cohorts.

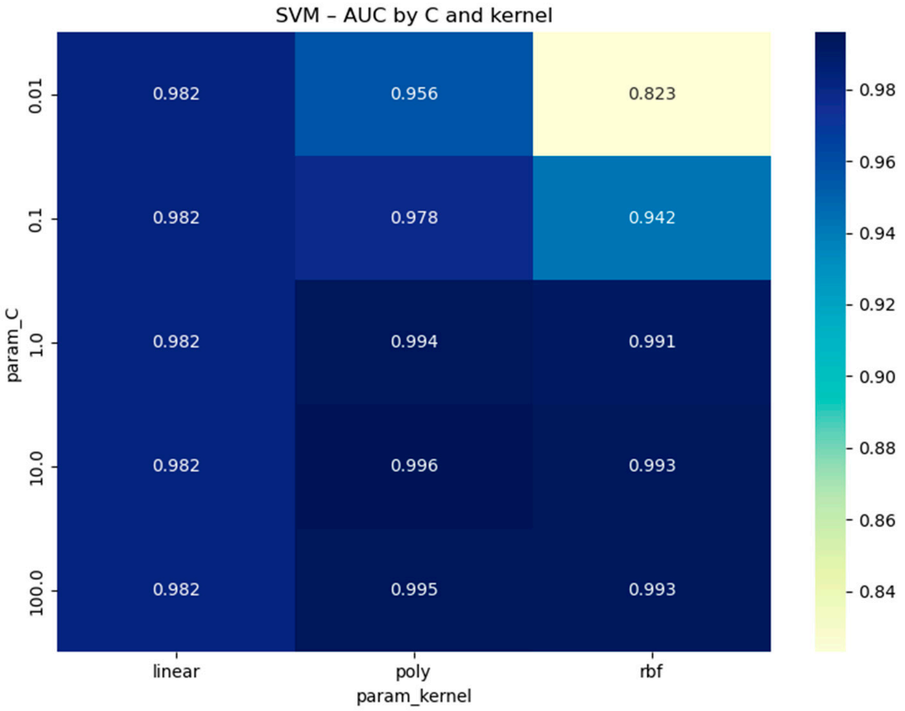

Figure S1. Heatmap of ROC-AUC values obtained using the optimal hyperparameter configuration for the Support Vector Machine classifier applied on the TCGA dataset.

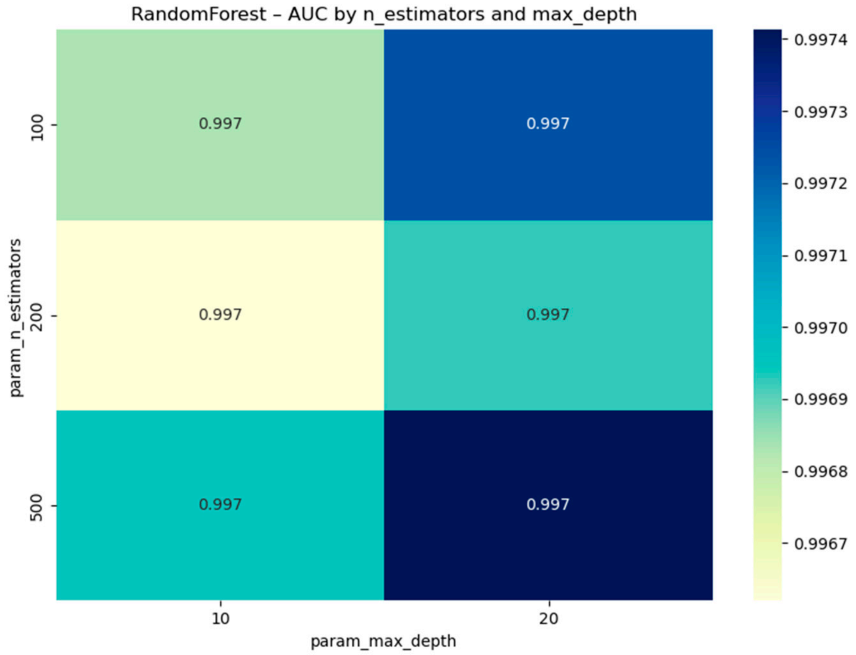

Figure S2. Heatmap of ROC-AUC values obtained using the optimal hyperparameter configuration for the Random Forest classifier applied on the TCGA dataset.

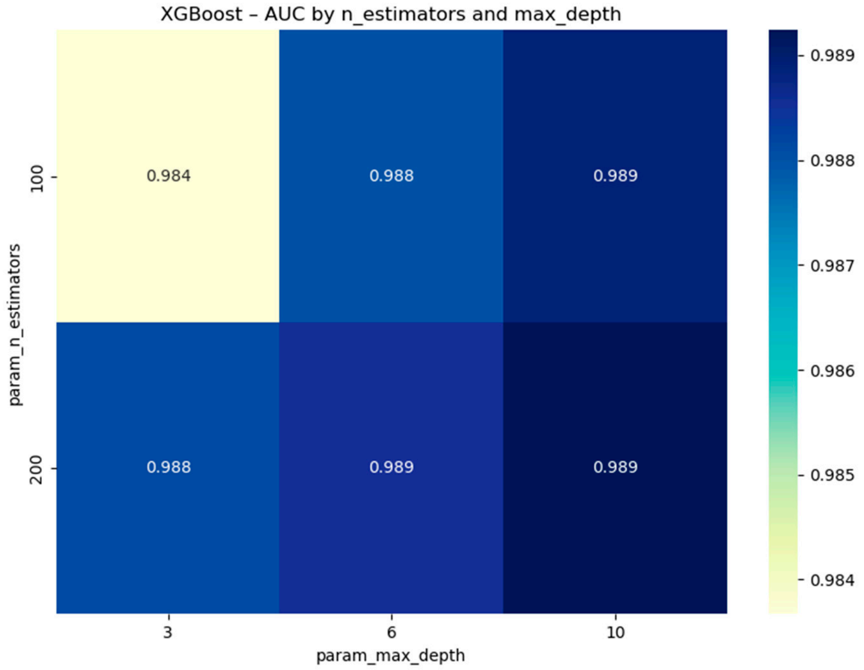

Figure S3. Heatmap of ROC-AUC values obtained using the optimal hyperparameter configuration for the XGBoost classifier applied on the TCGA dataset.

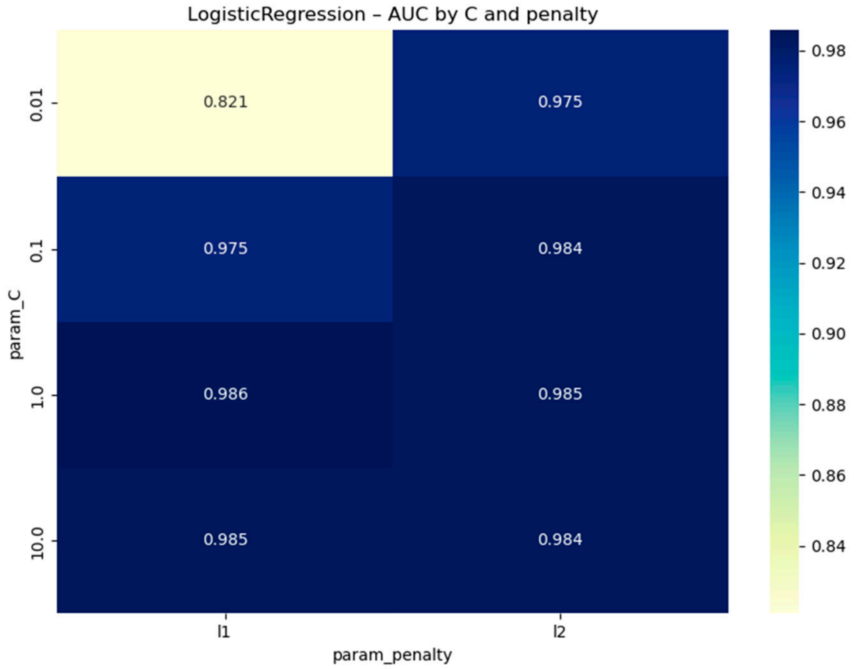

Figure S4. Heatmap of ROC-AUC values obtained using the optimal hyperparameter configuration for the Logistic Regression classifier applied on the TCGA dataset.

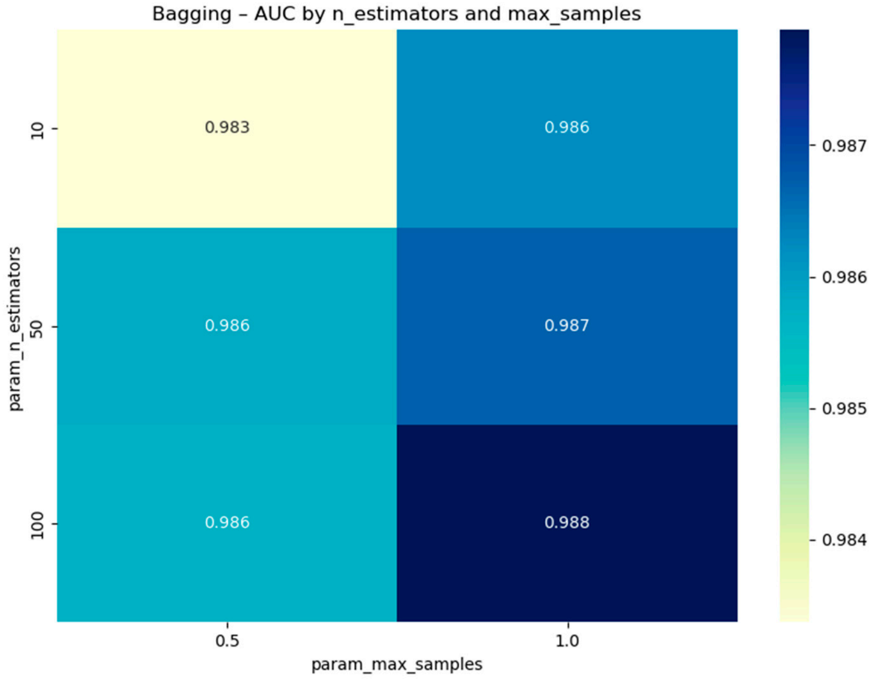

Figure S5. Heatmap of ROC-AUC values obtained using the optimal hyperparameter configuration for the Bagging classifier applied on the TCGA dataset.

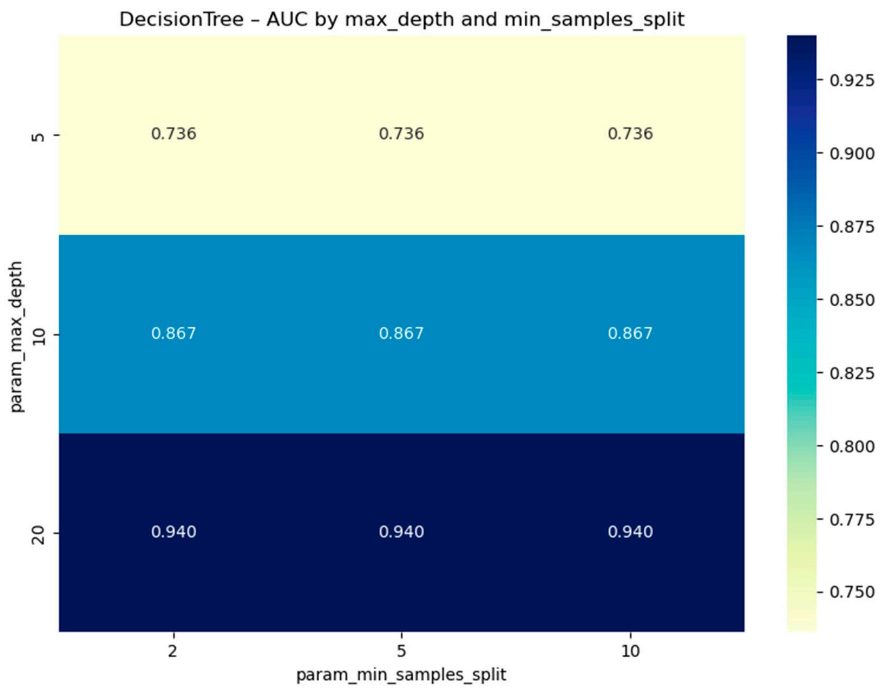

Figure S6. Heatmap of ROC-AUC values obtained using the optimal hyperparameter configuration for the DecisionTree classifier applied on the TCGA dataset.

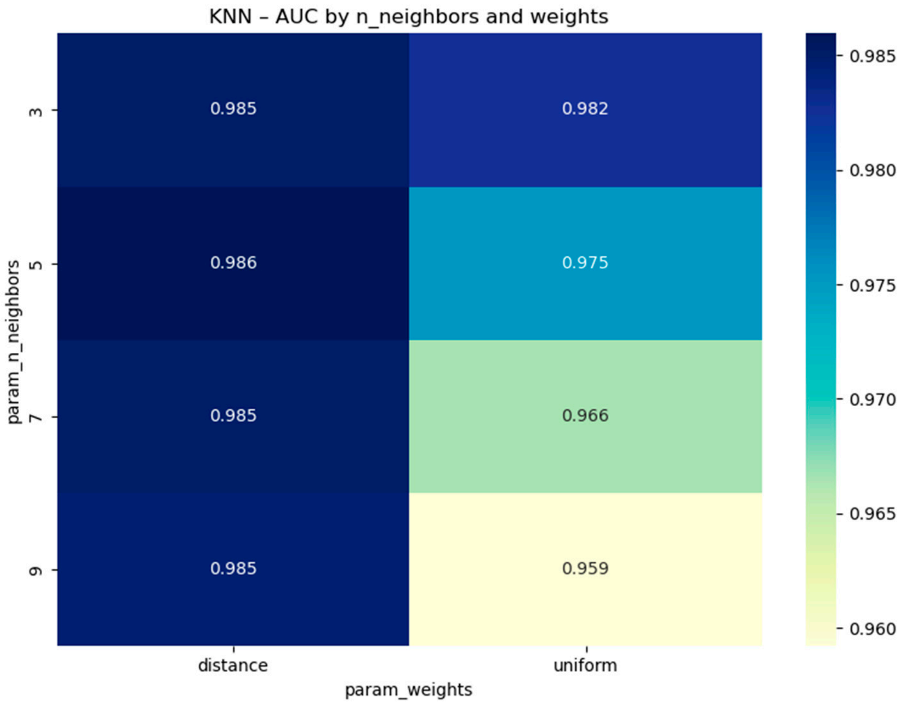

Figure S7. Heatmap of ROC-AUC values obtained using the optimal hyperparameter configuration for the k-Nearest Neighbors classifier applied on the TCGA dataset.

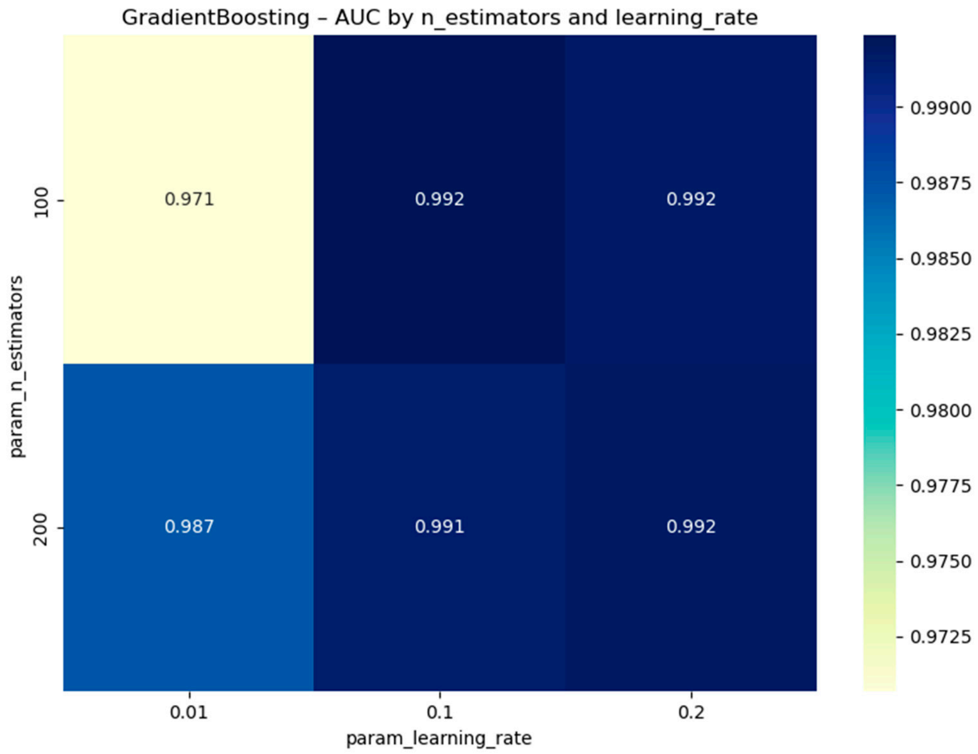

Figure S8. Heatmap of ROC-AUC values obtained using the optimal hyperparameter configuration for the Gradient Boosting classifier applied on the TCGA dataset.

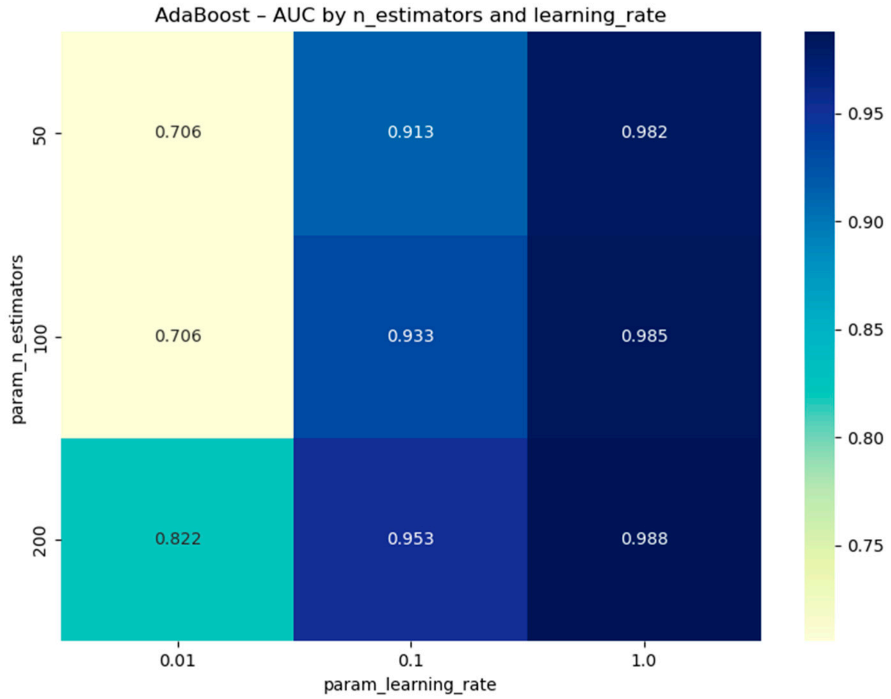

Figure S9. Heatmap of ROC-AUC values obtained using the optimal hyperparameter configuration for the AdaBoost classifier applied on the TCGA dataset.

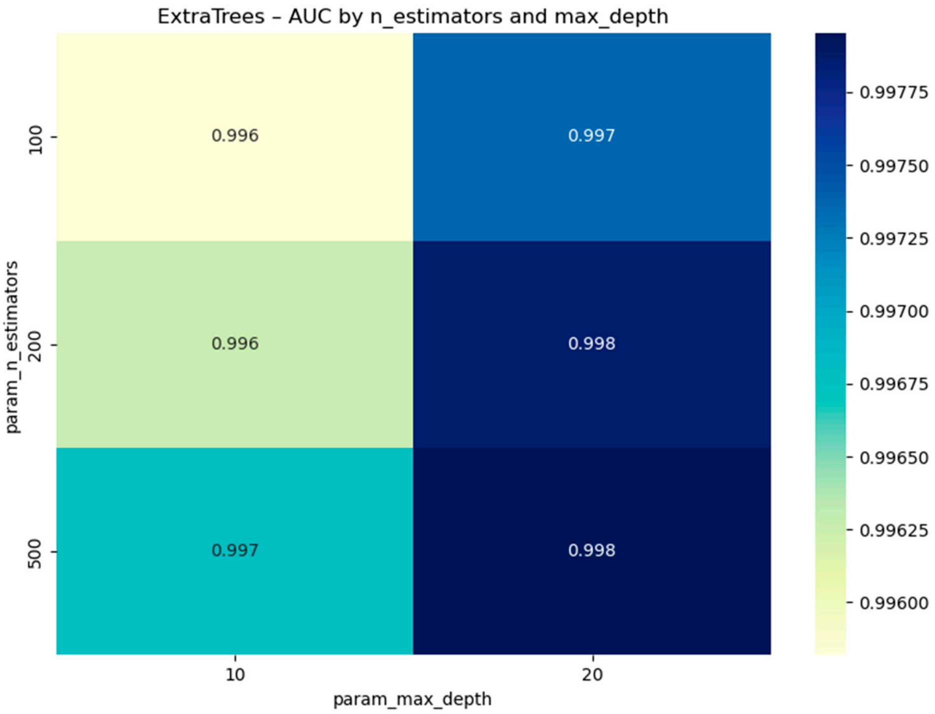

Figure S10. Heatmap of ROC-AUC values obtained using the optimal hyperparameter configuration for the ExtraTrees classifier applied on the TCGA dataset.

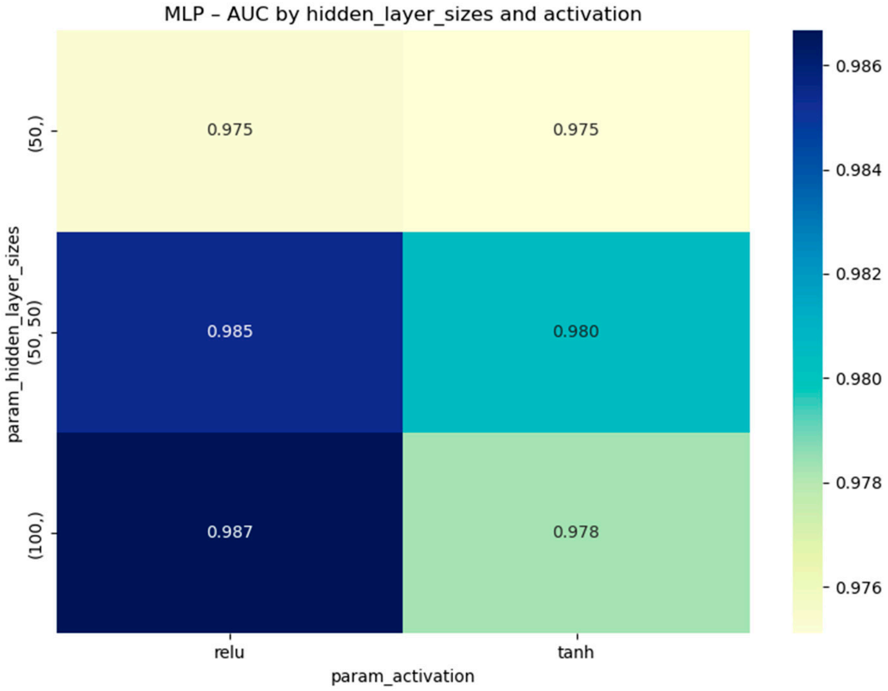

Figure S11. Heatmap of ROC-AUC values obtained using the optimal hyperparameter configuration for the Multilayer Perceptron classifier applied on the TCGA dataset.

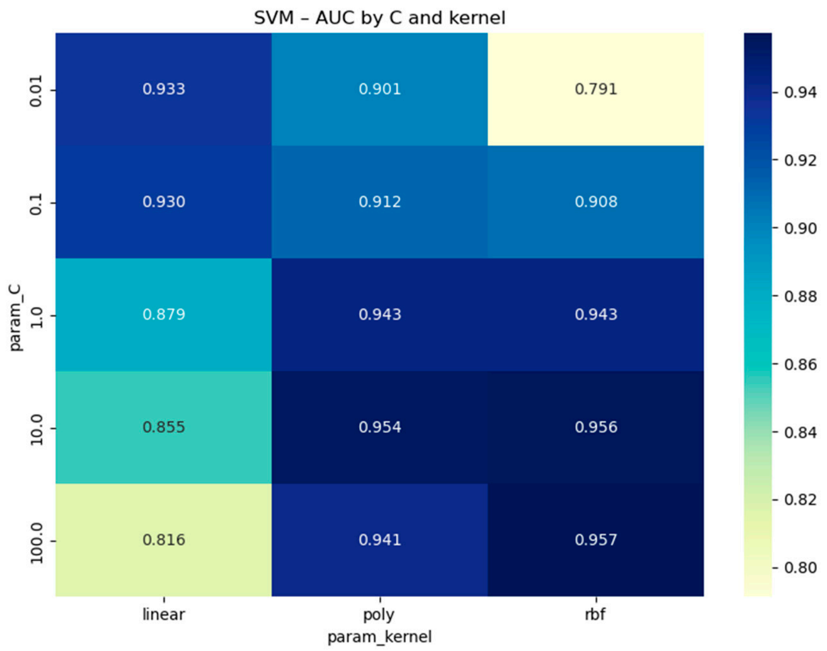

Figure S12. Heatmap of ROC-AUC values obtained using the optimal hyperparameter configuration for the Support Vector Machine classifier applied on the TCPA dataset.

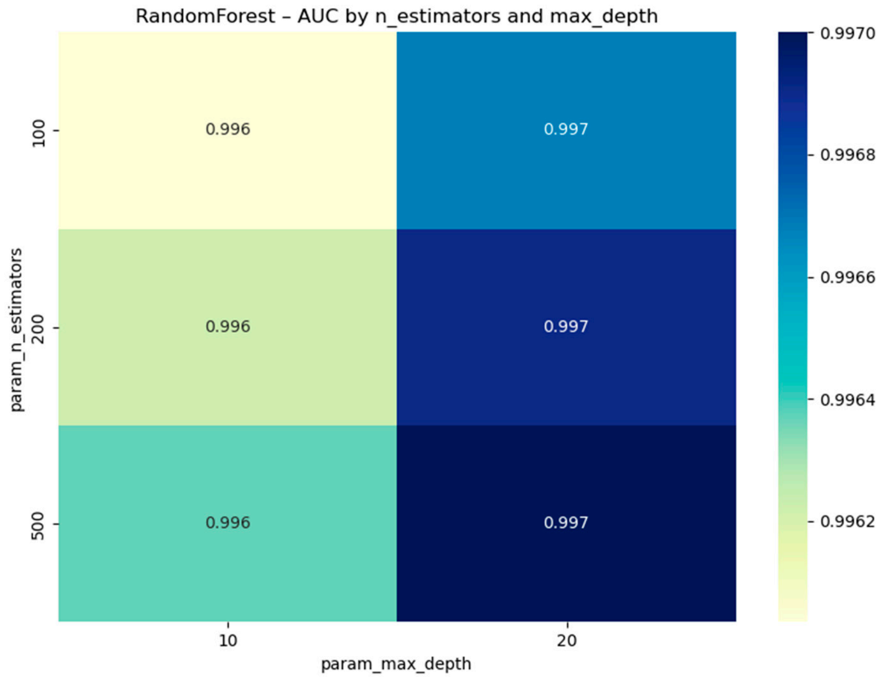

Figure S13. Heatmap of ROC-AUC values obtained using the optimal hyperparameter configuration for the Random Forest classifier applied on the TCPA dataset.

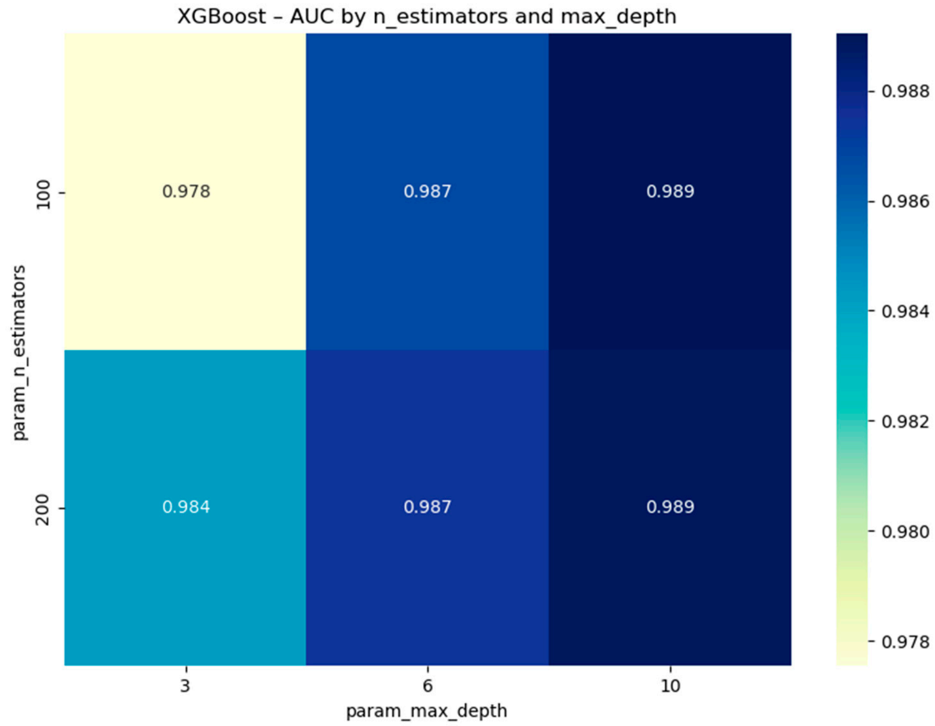

Figure S14. Heatmap of ROC-AUC values obtained using the optimal hyperparameter configuration for the XGBoost classifier applied on the TCPA dataset.

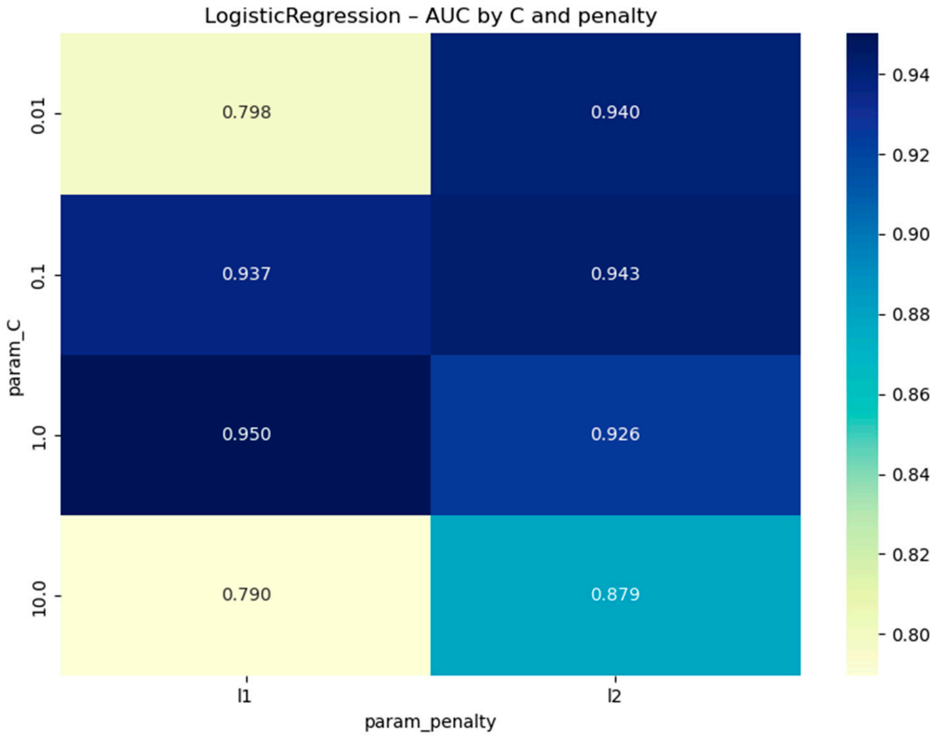

Figure S15. Heatmap of ROC-AUC values obtained using the optimal hyperparameter configuration for the Logistic Regression classifier applied on the TCPA dataset.

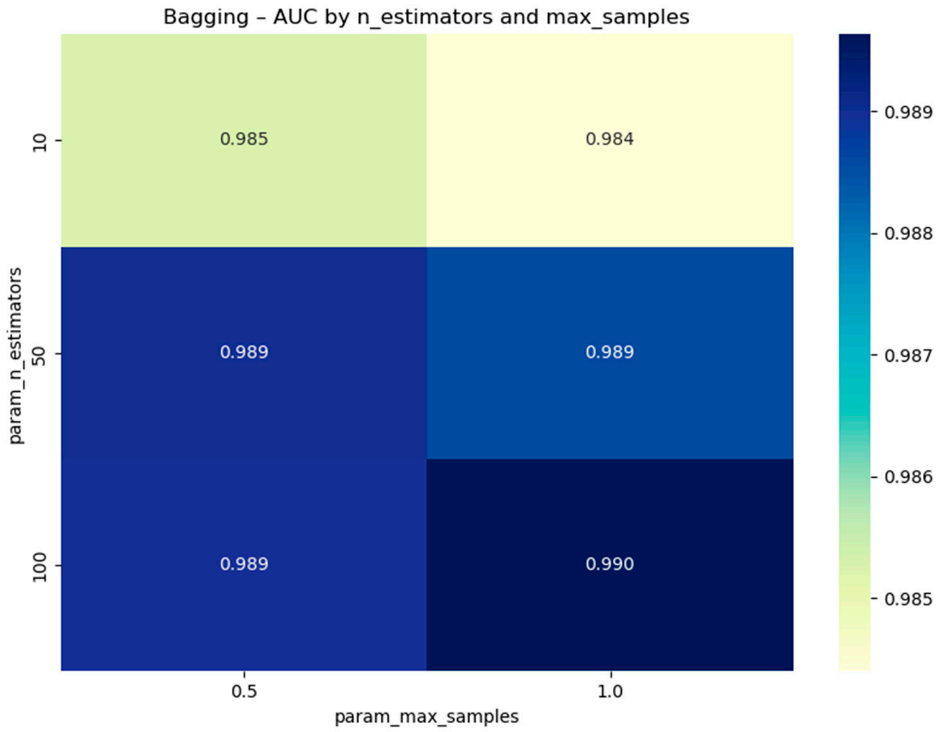

Figure S16. Heatmap of ROC-AUC values obtained using the optimal hyperparameter configuration for the Bagging classifier applied on the TCPA dataset.

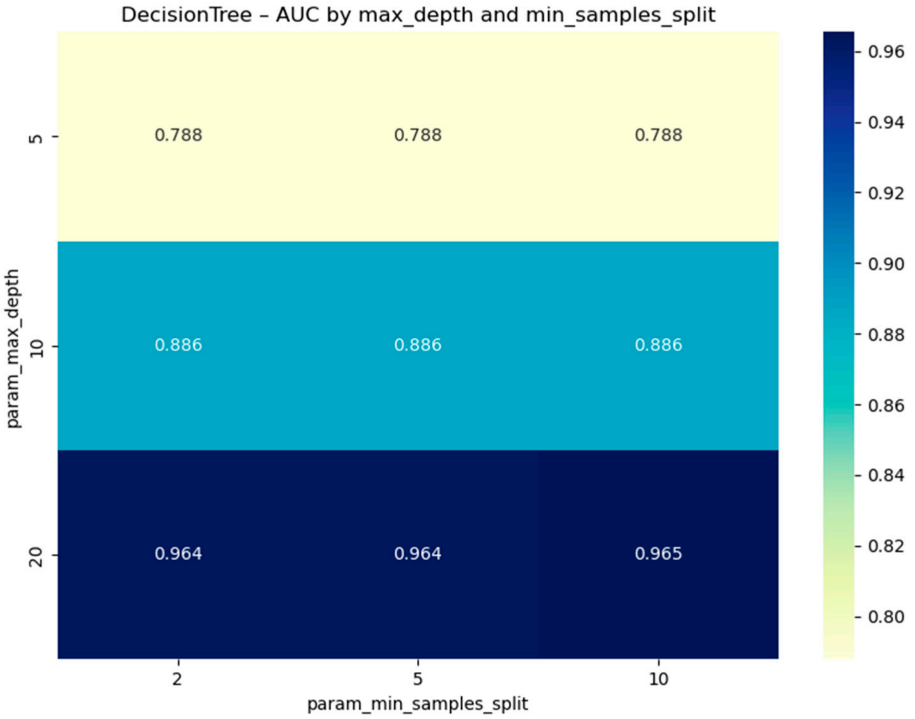

Figure S17. Heatmap of ROC-AUC values obtained using the optimal hyperparameter configuration for the DecisionTree classifier applied on the TCPA dataset.

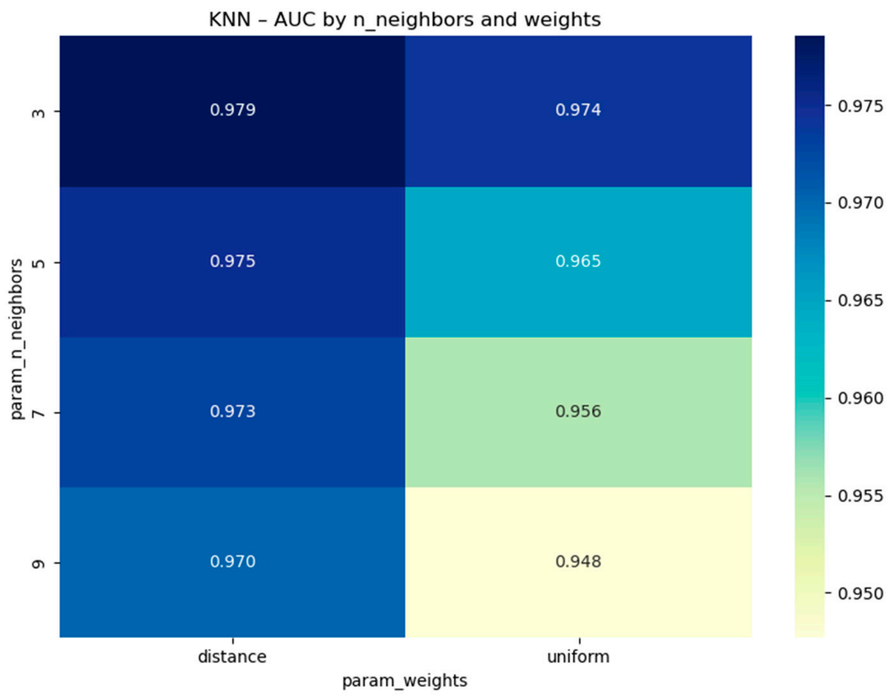

Figure S18. Heatmap of ROC-AUC values obtained using the optimal hyperparameter configuration for the k-Nearest Neighbors classifier applied on the TCPA dataset.

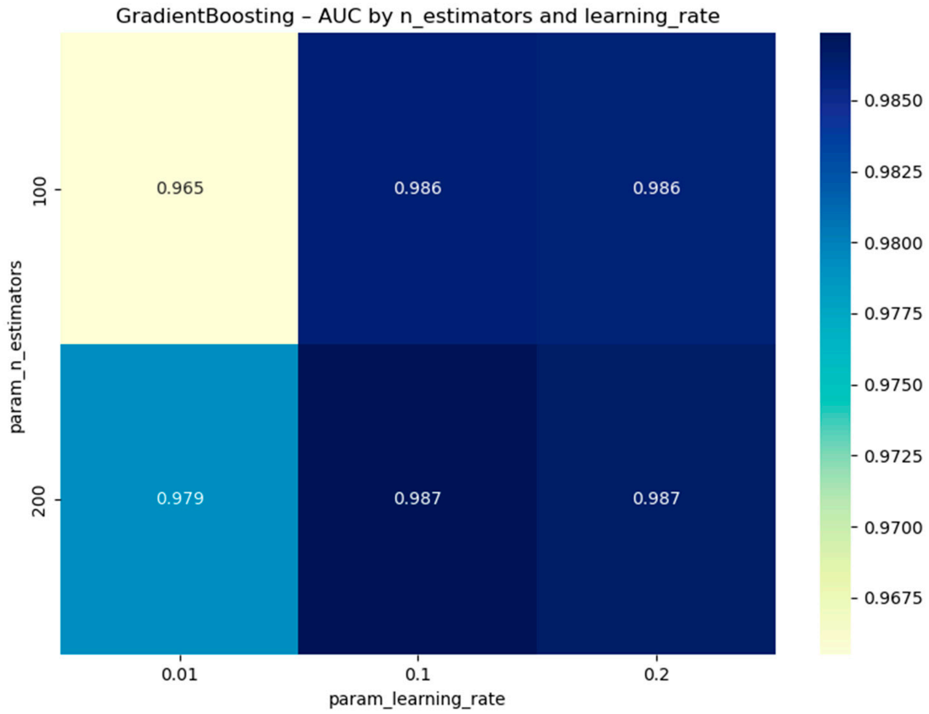

Figure S19. Heatmap of ROC-AUC values obtained using the optimal hyperparameter configuration for the Gradient Boosting classifier applied on the TCPA dataset.

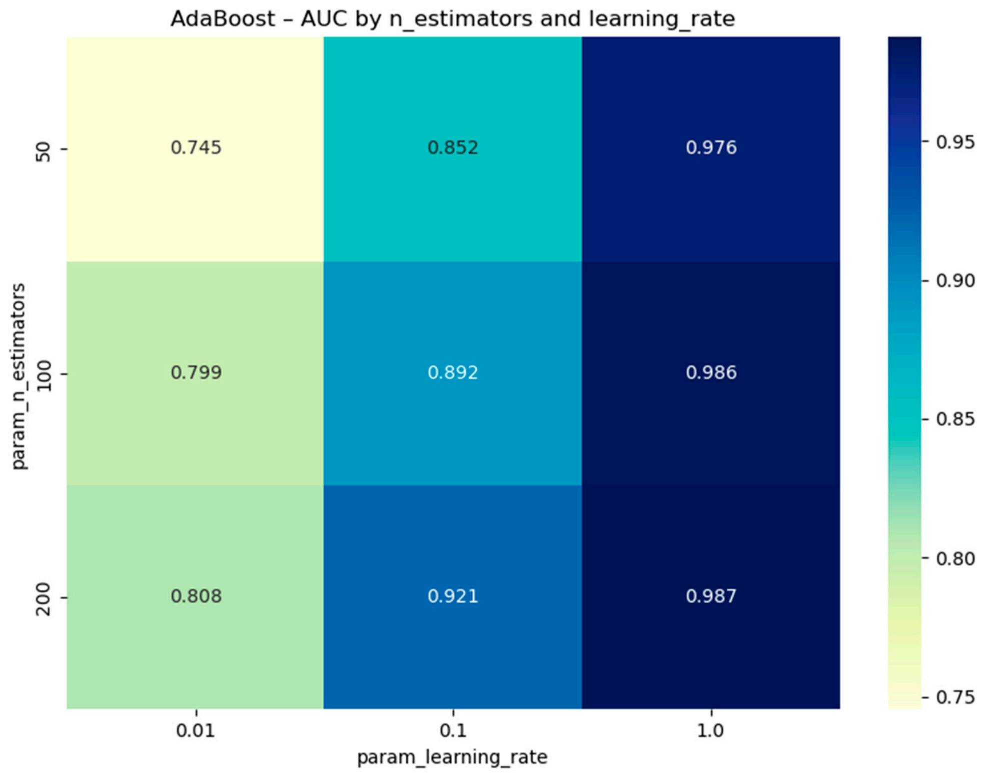

Figure S20. Heatmap of ROC-AUC values obtained using the optimal hyperparameter configuration for the AdaBoost classifier applied on the TCPA dataset.

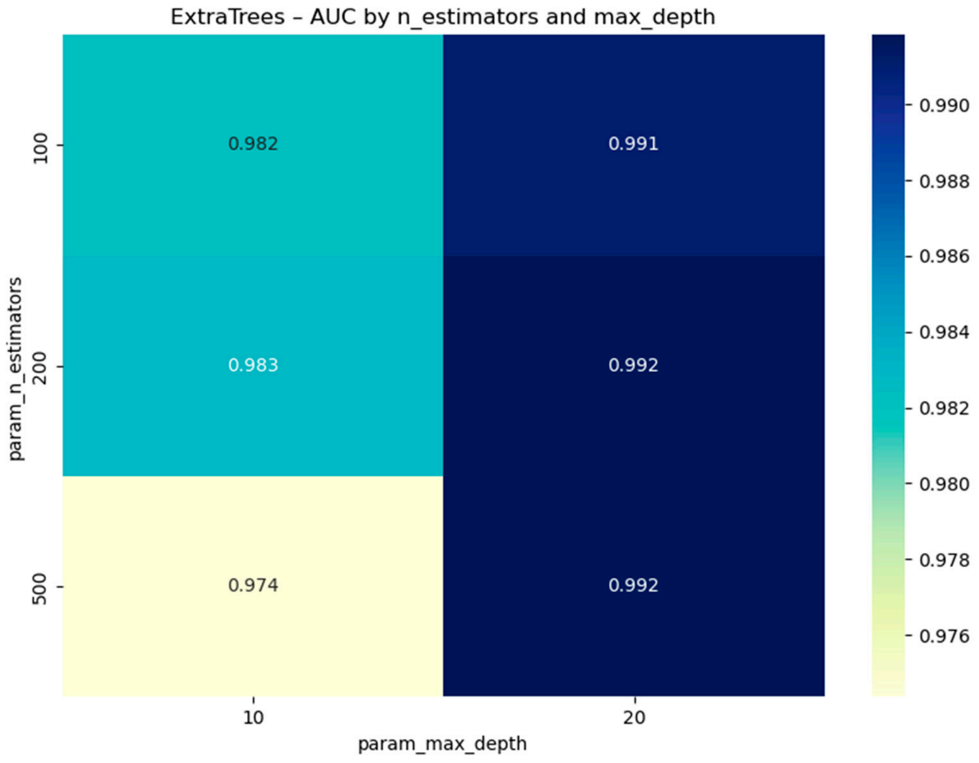

Figure S21. Heatmap of ROC-AUC values obtained using the optimal hyperparameter configuration for the ExtraTrees classifier applied on the TCPA dataset.

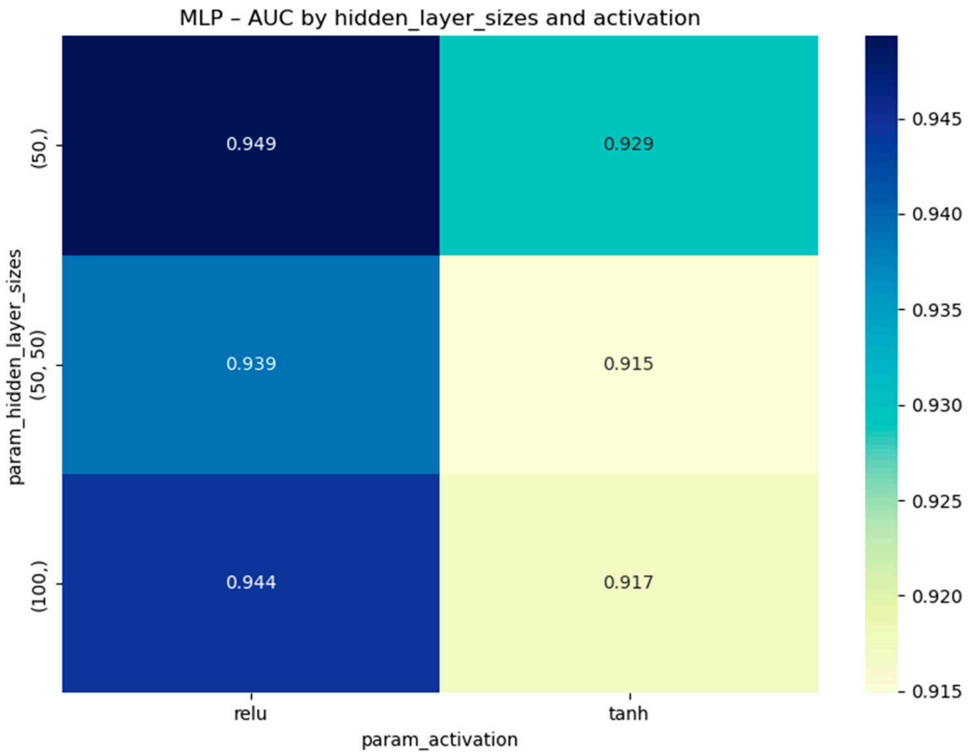

Figure S22. Heatmap of ROC-AUC values obtained using the optimal hyperparameter configuration for the Multilayer Perceptron classifier applied on the TCPA dataset.

Hyperparameter optimization was performed using a grid-search strategy within the cross-validation framework described in the Methods section. The hyperparameter search space and the optimal configurations identified for each classifier in the TCGA and TCPA datasets are summarized in Table S3. For GaussianNB and BernoulliNB, no grid-based hyperparameter tuning was performed, and default model settings were retained. When multiple configurations achieved equivalent ROC-AUC values after rounding, all equivalent optimal configurations were reported.

Table S3. Hyperparameter search space and optimal configurations identified for each machine learning algorithm. When multiple configurations achieved the same ROC-AUC value after rounding to three decimal places, all equivalent optimal configurations are reported.

**Algorithm**	**Optimization method**	**Hyperparameters explored**	**Optimal TCGA configuration**	**Optimal TCPA configuration**
SVM	Grid search	C, kernel	C = 10; kernel = poly	C = 100; kernel = rbf
RF	Grid search	n_estimators, max_depth	n_estimators = 500; max_depth = 20	n_estimators = 500; max_depth = 20
XGBoost	Grid search	n_estimators, max_depth	n_estimators = 200; max_depth = 10	n_estimators = 100/200; max_depth = 10
LR	Grid search	C, penalty	C = 1; penalty = l1	C = 1; penalty = l1
Bagging	Grid search	n_estimators, max_samples	n_estimators = 100; max_samples = 1.0	n_estimators = 100; max_samples = 1.0
Decision Tree	Grid search	max_depth, min_samples_split	max_depth = 20; min_samples_split = 2/5/10	max_depth = 20; min_samples_split = 10
GaussianNB	Not applicable / default settings	—	Default configuration	Default configuration
BernoulliNB	Not applicable / default settings	—	Default configuration	Default configuration
KNN	Grid search	n_neighbors, weights	n_neighbors = 5; weights = distance	n_neighbors = 3; weights = distance
Gradient Boosting	Grid search	n_estimators, learning_rate	n_estimators = 100/200; learning_rate = 0.2	n_estimators = 200; learning_rate = 0.1/0.2

AdaBoost	Grid search	n_estimators, learning_rate	n_estimators = 200; learning_rate = 1.0	n_estimators = 200; learning_rate = 1.0
ExtraTrees	Grid search	n_estimators, max_depth	n_estimators = 200/500; max_depth = 20	n_estimators = 200/500; max_depth = 20
MLP	Grid search	hidden_layer_sizes, activation	hidden_layer_sizes = 100; activation = relu	hidden_layer_sizes = 50; activation = relu

Supplementary Figures S23–48 illustrate the feature importance profiles obtained for each of the thirteen supervised machine learning algorithms applied in this study. For each classifier, the top 20 most informative proteomic features were identified based on model-specific importance measures and are visualized to highlight algorithm-dependent patterns. These analyses were performed separately for the TCPA and TCGA datasets, allowing assessment of dataset-specific and model-specific feature relevance.

The Supplementary Figures provide a detailed overview of how individual machine learning models prioritize proteomic features and support the consensus-based feature selection strategy adopted in the main text. While algorithm-specific rankings capture model-dependent signals, their aggregation into a global ranking enables the identification of robust and consistently informative biomarkers across multiple learning paradigms.

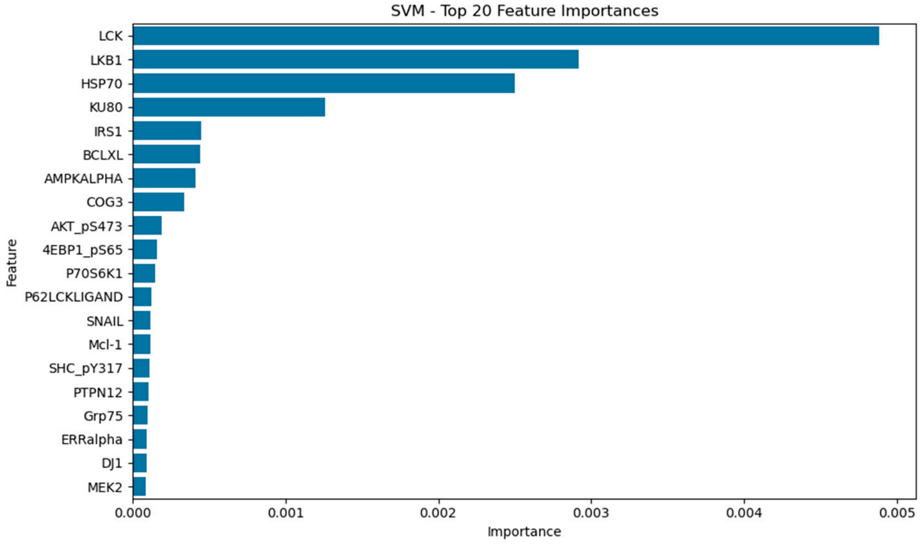

Figure S23. Feature importance analysis for the Support Vector Machine classifier applied to the TCGA dataset. The plot reports the top 20 proteomic features ranked according to model-specific importance scores. Feature importance values reflect the relative contribution of each protein to the prediction of therapeutic response. These algorithm-specific rankings were used as input for the consensus-based global feature selection strategy described in the main text.

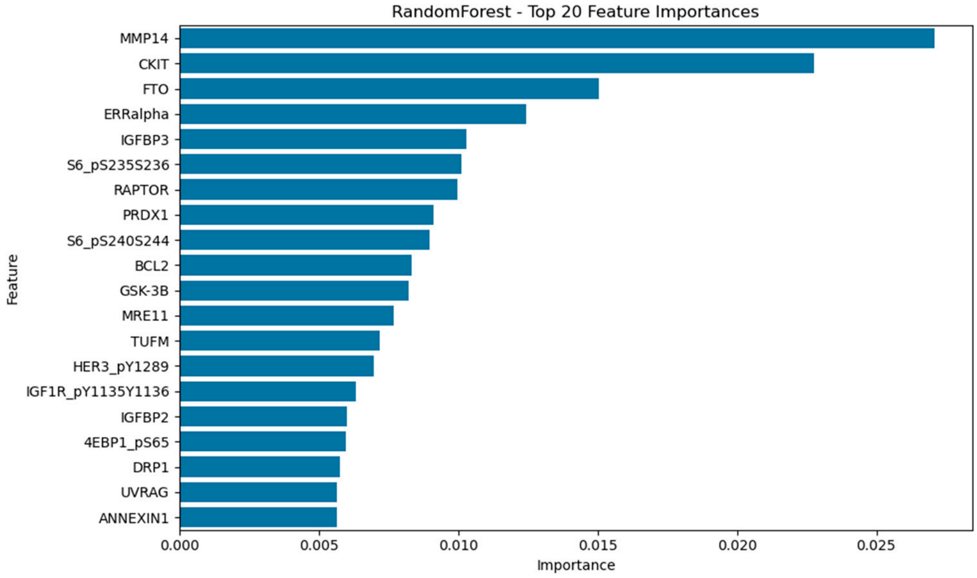

Figure S24. Feature importance analysis for the Random Forest classifier applied to the TCGA dataset. The plot reports the top 20 proteomic features ranked according to model-specific importance scores. Feature importance values reflect the relative contribution of each protein to the prediction of therapeutic response. These algorithm-specific rankings were used as input for the consensus-based global feature selection strategy described in the main text.

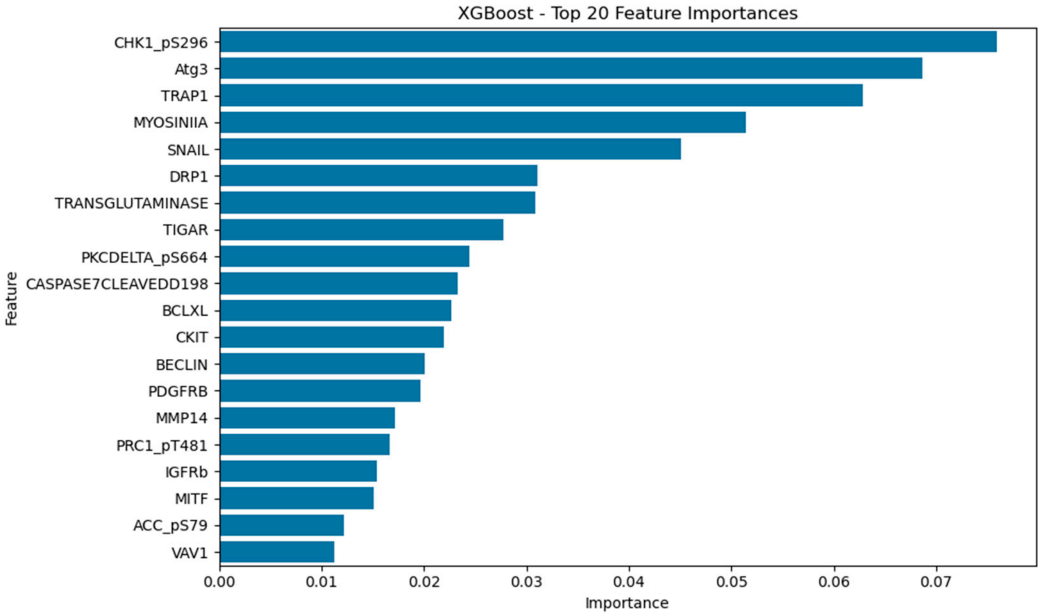

Figure S25. Feature importance analysis for the XGBoost classifier applied to the TCGA dataset. The plot reports the top 20 proteomic features ranked according to model-specific importance scores. Feature importance values reflect the relative contribution of each protein to the prediction of therapeutic response. These algorithm-specific rankings were used as input for the consensus-based global feature selection strategy described in the main text.

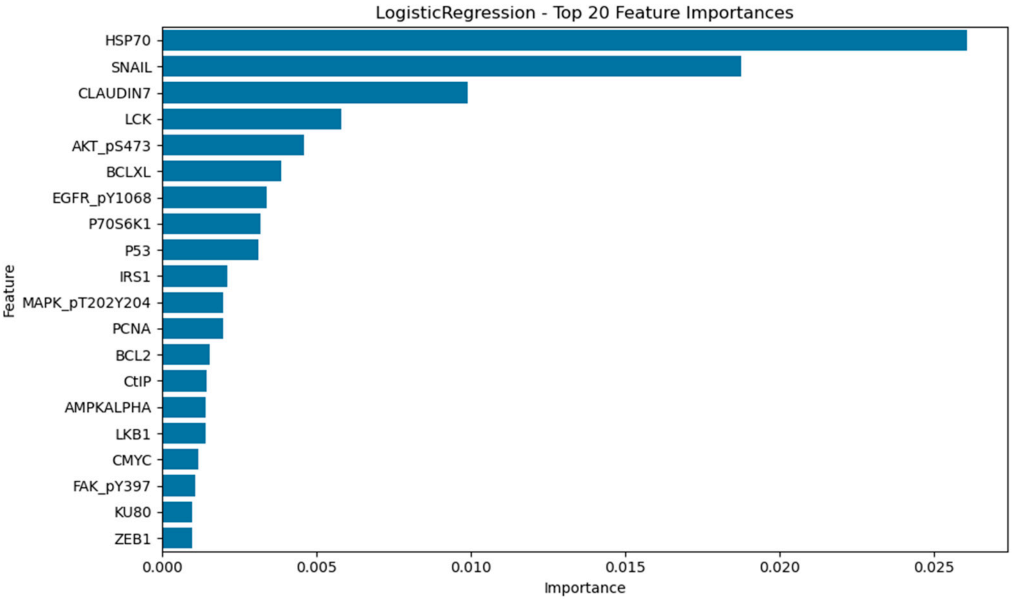

Figure S26. Feature importance analysis for the Logistic Regression classifier applied to the TCGA dataset. The plot reports the top 20 proteomic features ranked according to model-specific importance scores. Feature importance values reflect the relative contribution of each protein to the prediction of therapeutic response. These algorithm-specific rankings were used as input for the consensus-based global feature selection strategy described in the main text.

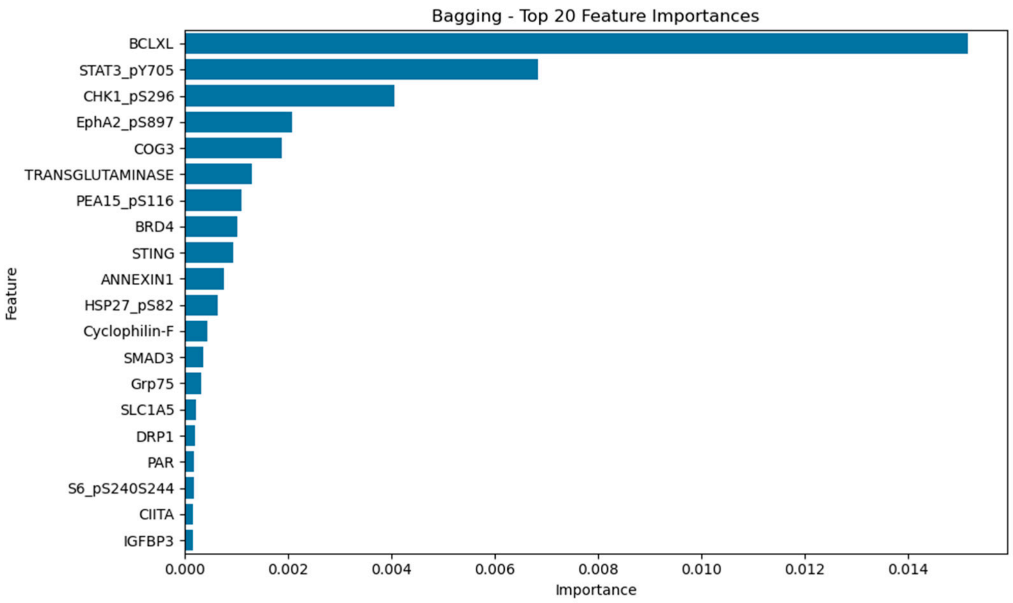

Figure S27. Feature importance analysis for the Bagging classifier applied to the TCGA dataset. The plot reports the top 20 proteomic features ranked according to model-specific importance scores. Feature importance values reflect the relative contribution of each protein to the prediction of therapeutic response. These algorithm-specific rankings were used as input for the consensus-based global feature selection strategy described in the main text.

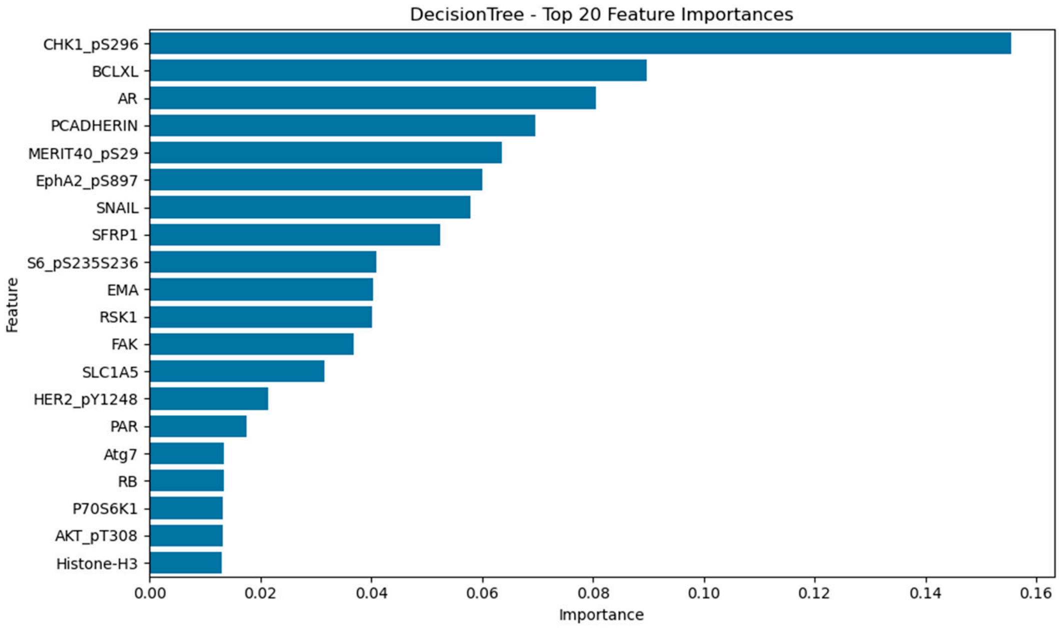

Figure S28. Feature importance analysis for the DecisionTree classifier applied to the TCGA dataset. The plot reports the top 20 proteomic features ranked according to model-specific importance scores. Feature importance values reflect the relative contribution of each protein to the prediction of therapeutic response. These algorithm-specific rankings were used as input for the consensus-based global feature selection strategy described in the main text.

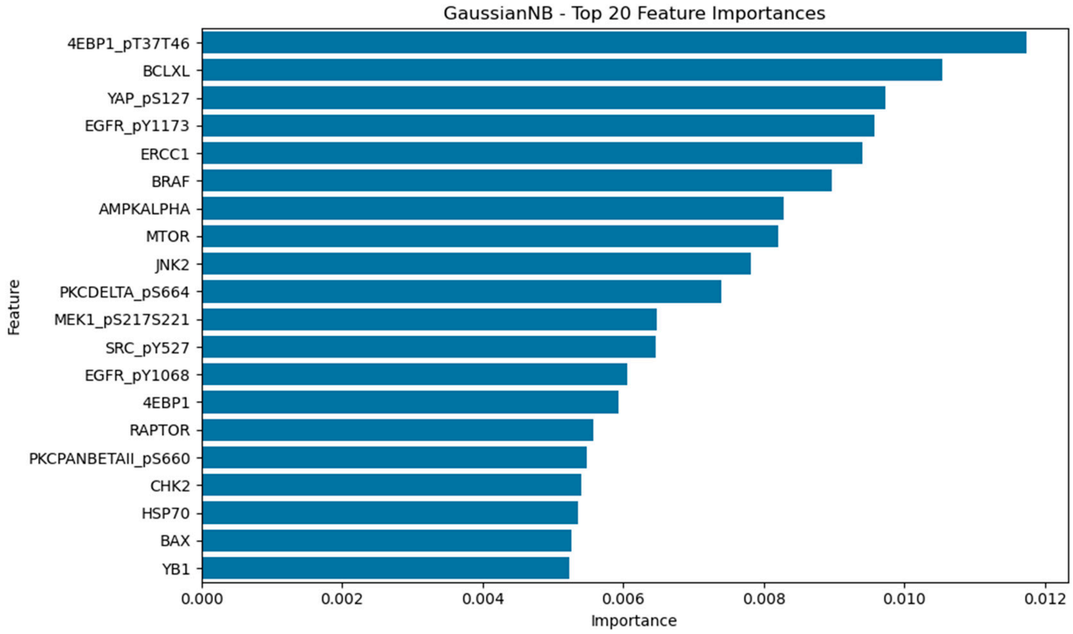

Figure S29. Feature importance analysis for the GaussianNB classifier applied to the TCGA dataset. The plot reports the top 20 proteomic features ranked according to model-specific importance scores. Feature importance values reflect the relative contribution of each protein to the prediction of therapeutic response. These algorithm-specific rankings were used as input for the consensus-based global feature selection strategy described in the main text.

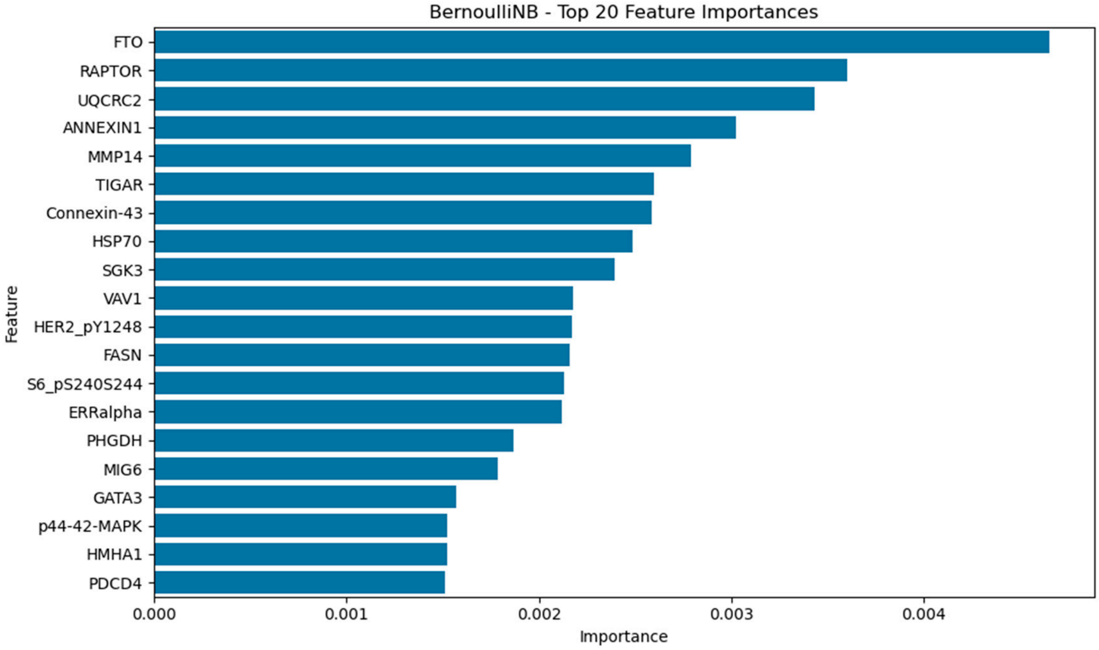

Figure S30. Feature importance analysis for the BernoulliNB classifier applied to the TCGA dataset. The plot reports the top 20 proteomic features ranked according to model-specific importance scores. Feature importance values reflect the relative contribution of each protein to the prediction of therapeutic response. These algorithm-specific rankings were used as input for the consensus-based global feature selection strategy described in the main text.

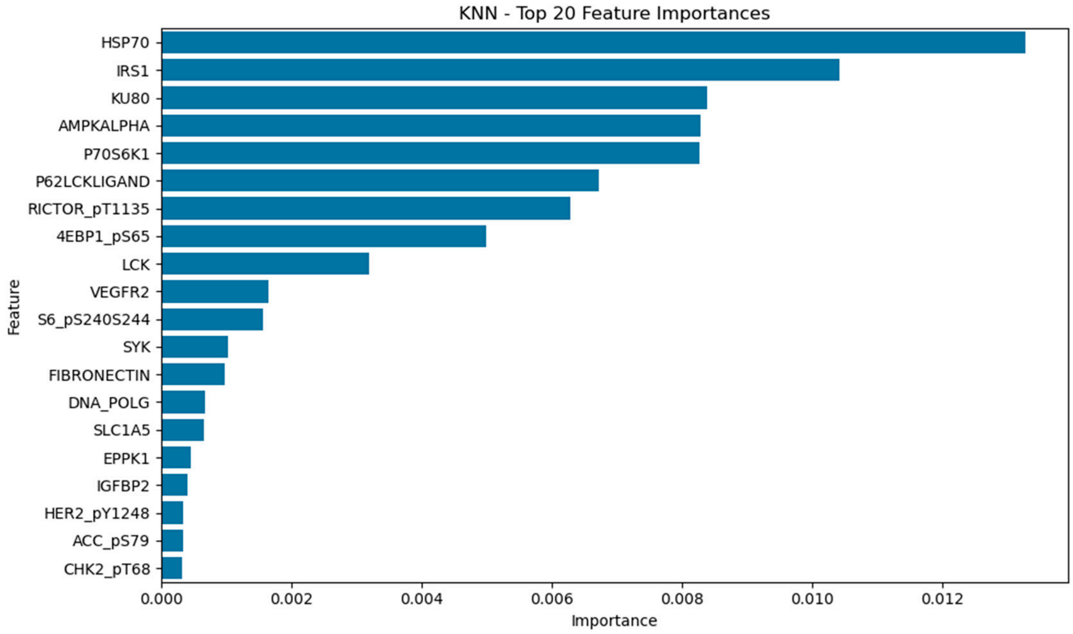

Figure S31. Feature importance analysis for the k-Nearest Neighbors classifier applied to the TCGA dataset. The plot reports the top 20 proteomic features ranked according to model-specific importance scores. Feature importance values reflect the relative contribution of each protein to the prediction of therapeutic response. These algorithm-specific rankings were used as input for the consensus-based global feature selection strategy described in the main text.

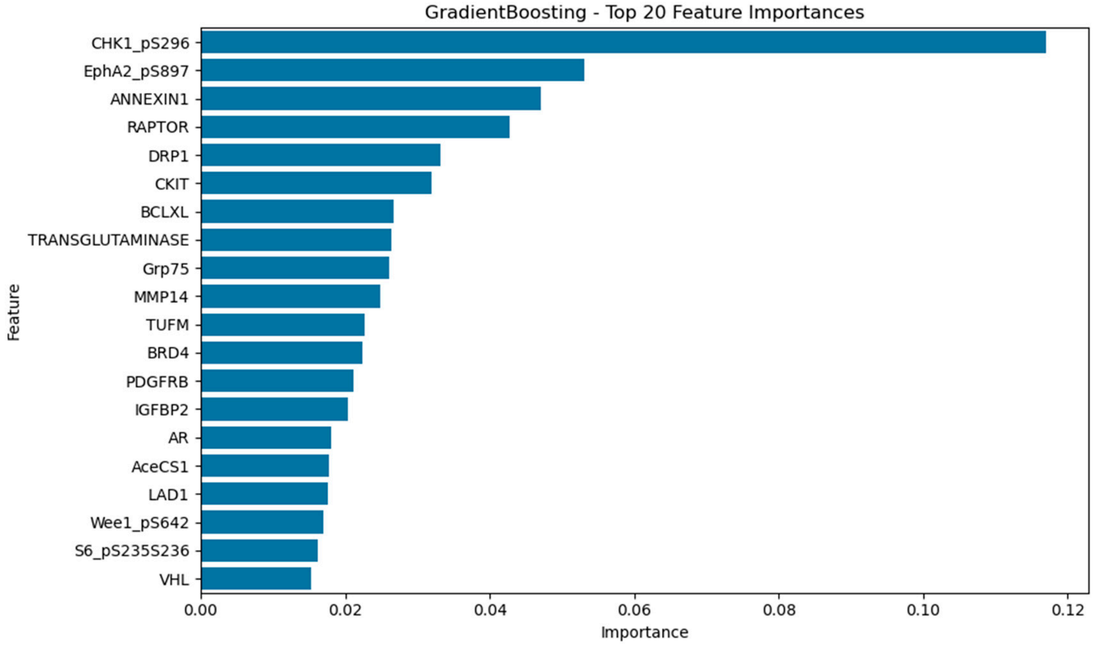

Figure S32. Feature importance analysis for the Gradient Boosting classifier applied to the TCGA dataset. The plot reports the top 20 proteomic features ranked according to model-specific importance scores. Feature importance values reflect the relative contribution of each protein to the prediction of therapeutic response. These algorithm-specific rankings were used as input for the consensus-based global feature selection strategy described in the main text.

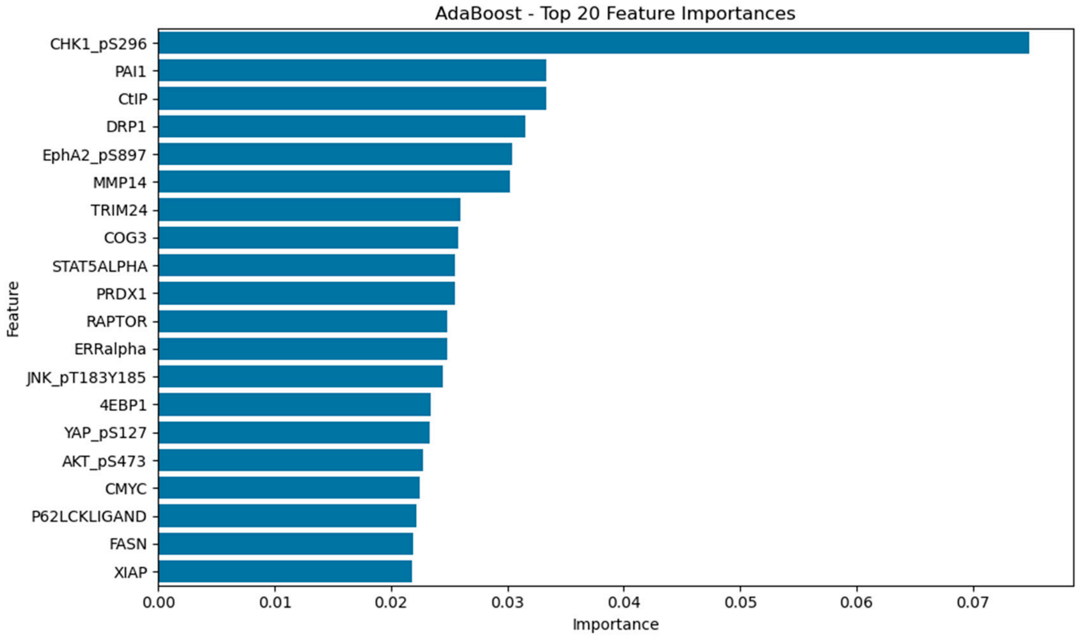

Figure S33. Feature importance analysis for the AdaBoost classifier applied to the TCGA dataset. The plot reports the top 20 proteomic features ranked according to model-specific importance scores. Feature importance values reflect the relative contribution of each protein to the prediction of therapeutic response. These algorithm-specific rankings were used as input for the consensus-based global feature selection strategy described in the main text.

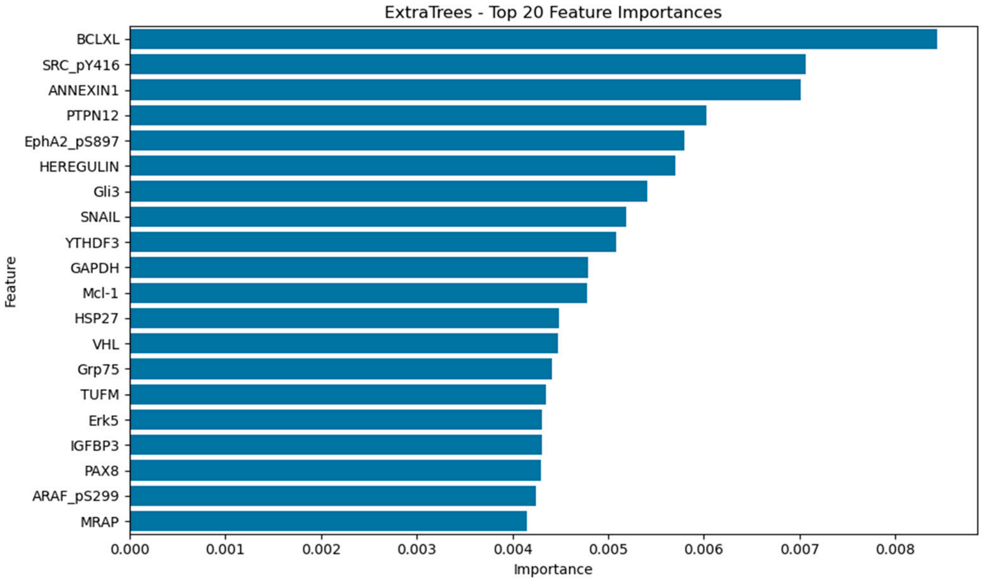

Figure S34. Feature importance analysis for the ExtraTrees classifier applied to the TCGA dataset. The plot reports the top 20 proteomic features ranked according to model-specific importance scores. Feature importance values reflect the relative contribution of each protein to the prediction of therapeutic response. These algorithm-specific rankings were used as input for the consensus-based global feature selection strategy described in the main text.

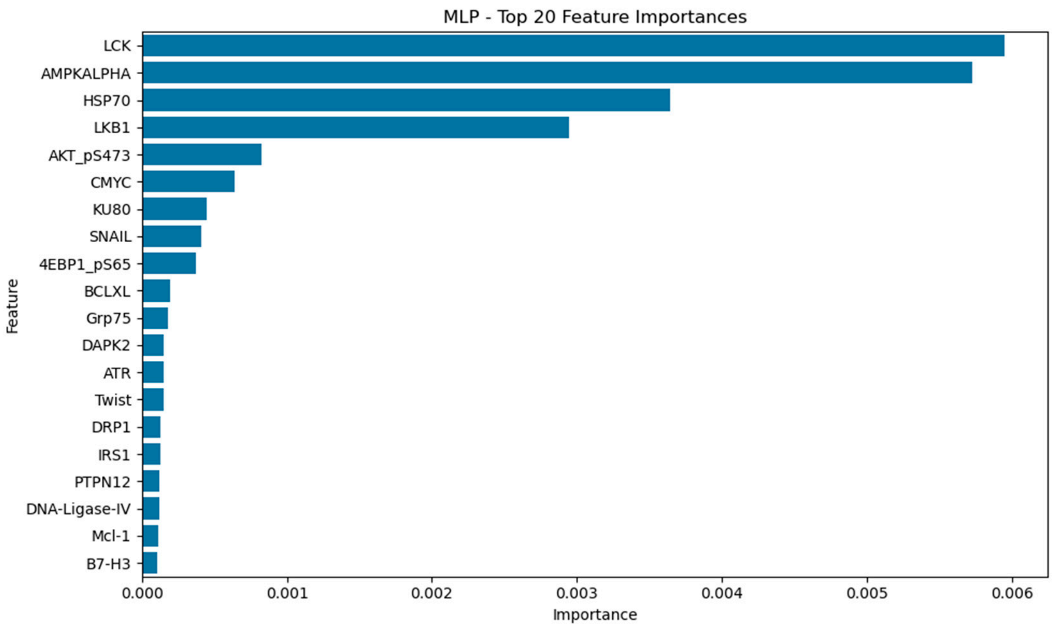

Figure S35. Feature importance analysis for the Multilayer Perceptron classifier applied to the TCGA dataset. The plot reports the top 20 proteomic features ranked according to model-specific importance scores. Feature importance values reflect the relative contribution of each protein to the prediction of therapeutic response. These algorithm-specific rankings were used as input for the consensus-based global feature selection strategy described in the main text.

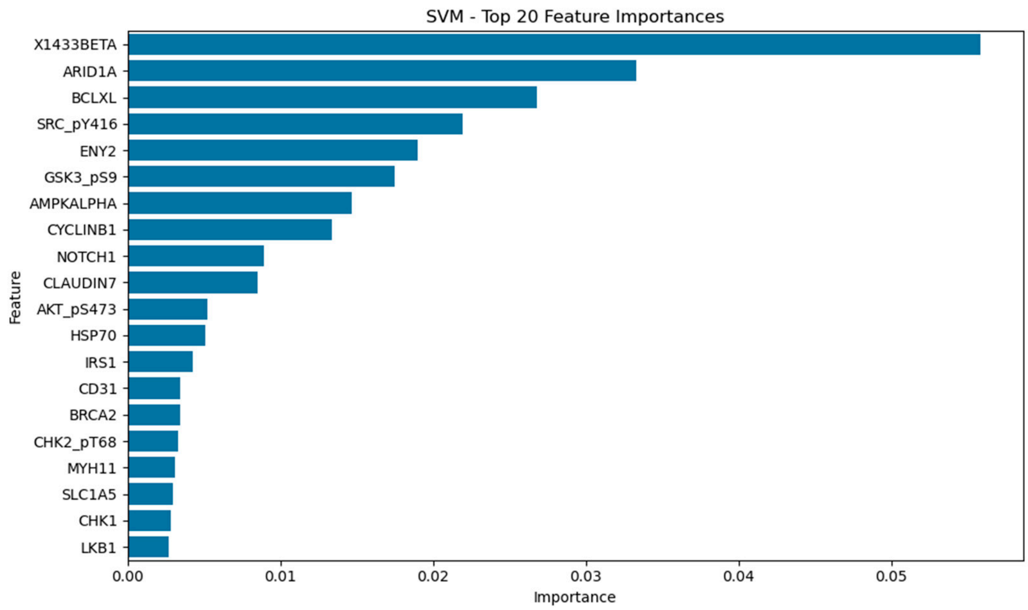

Figure S36. Feature importance analysis for the Support Vector Machine classifier applied to the TCPA dataset. The plot reports the top 20 proteomic features ranked according to model-specific importance scores. Feature importance values reflect the relative contribution of each protein to the prediction of therapeutic response. These algorithm-specific rankings were used as input for the consensus-based global feature selection strategy described in the main text.

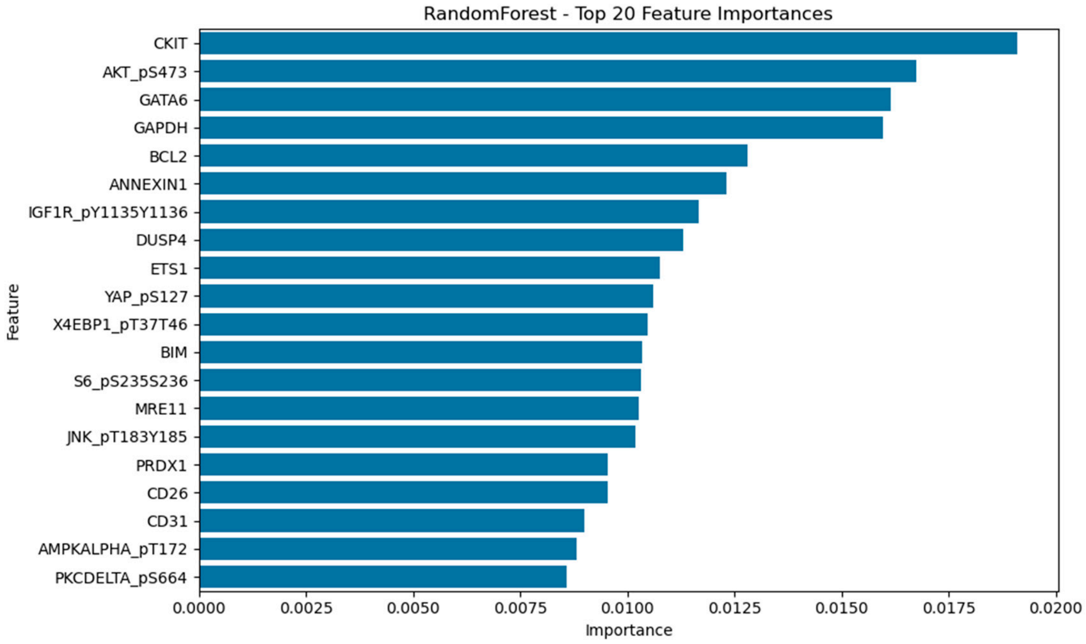

Figure S37. Feature importance analysis for the Random Forest classifier applied to the TCPA dataset. The plot reports the top 20 proteomic features ranked according to model-specific importance scores. Feature importance values reflect the relative contribution of each protein to the prediction of therapeutic response. These algorithm-specific rankings were used as input for the consensus-based global feature selection strategy described in the main text.

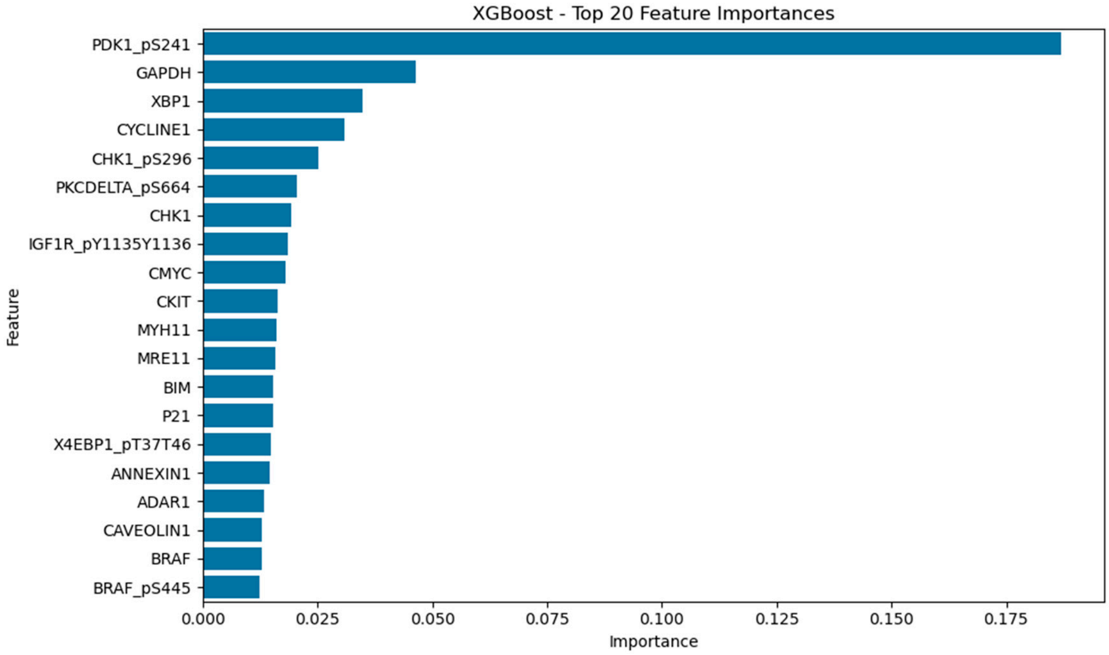

Figure S38. Feature importance analysis for the XGBoost classifier applied to the TCPA dataset. The plot reports the top 20 proteomic features ranked according to model-specific importance scores. Feature importance values reflect the relative contribution of each protein to the prediction of therapeutic response. These algorithm-specific rankings were used as input for the consensus-based global feature selection strategy described in the main text.

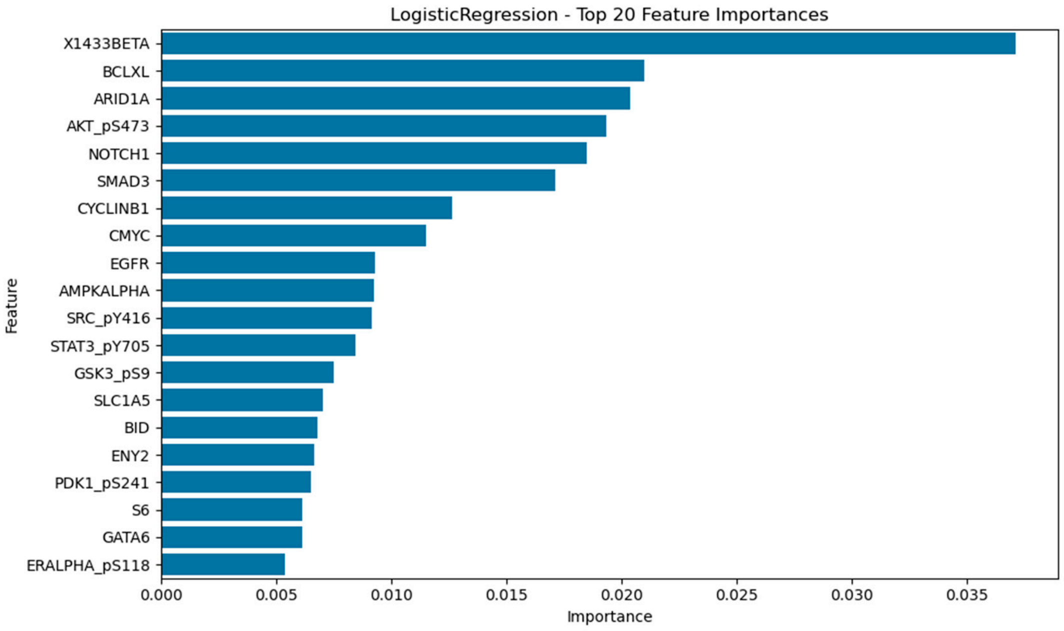

Figure S39. Feature importance analysis for the Logistic Regression classifier applied to the TCPA dataset. The plot reports the top 20 proteomic features ranked according to model-specific importance scores. Feature importance values reflect the relative contribution of each protein to the prediction of therapeutic response. These algorithm-specific rankings were used as input for the consensus-based global feature selection strategy described in the main text.

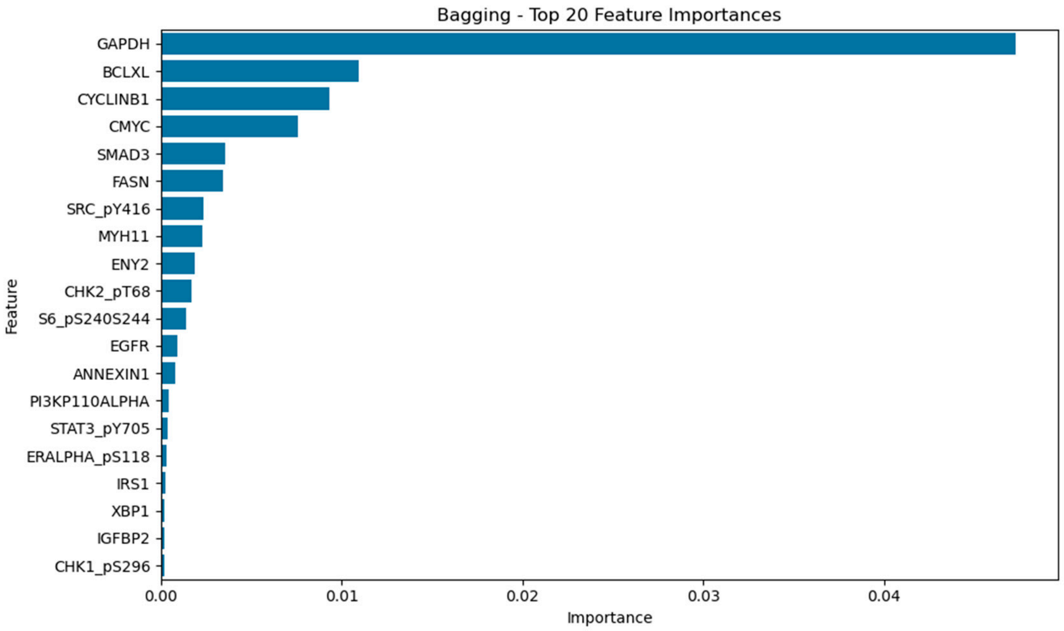

Figure S40. Feature importance analysis for the Bagging classifier applied to the TCPA dataset. The plot reports the top 20 proteomic features ranked according to model-specific importance scores. Feature importance values reflect the relative contribution of each protein to the prediction of therapeutic response. These algorithm-specific rankings were used as input for the consensus-based global feature selection strategy described in the main text.

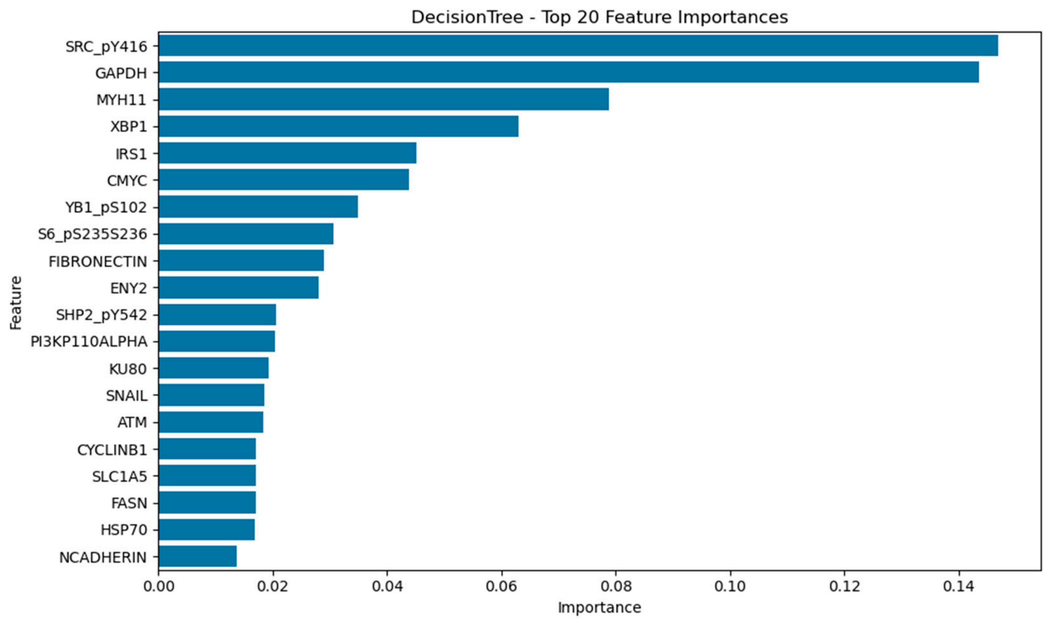

Figure S41. Feature importance analysis for the DecisionTree classifier applied to the TCPA dataset. The plot reports the top 20 proteomic features ranked according to model-specific importance scores. Feature importance values reflect the relative contribution of each protein to the prediction of therapeutic response. These algorithm-specific rankings were used as input for the consensus-based global feature selection strategy described in the main text.

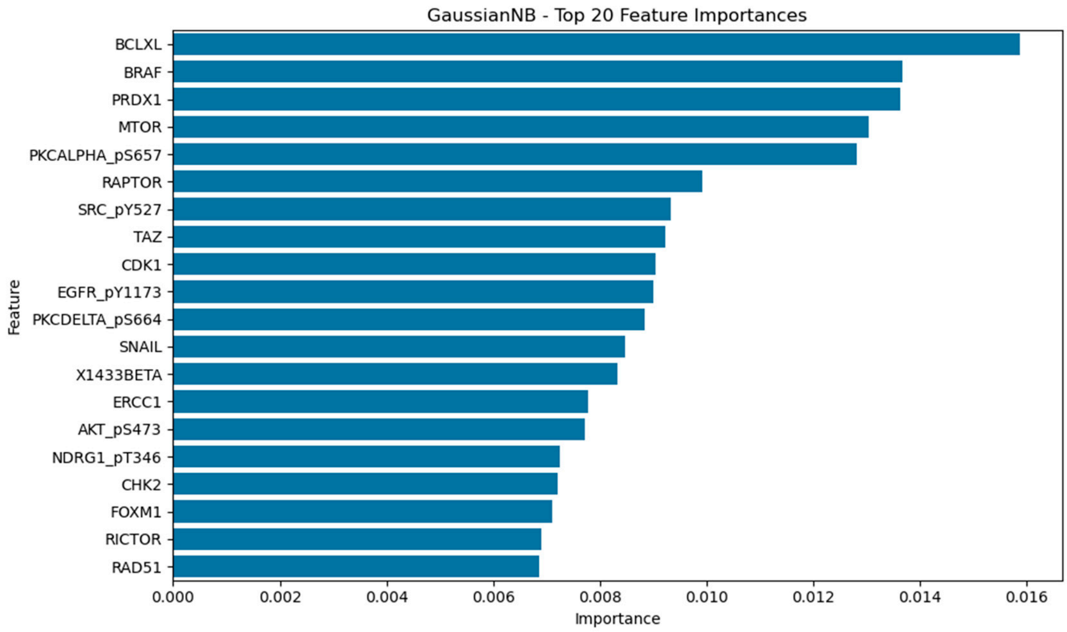

Figure S42. Feature importance analysis for the GaussianNB classifier applied to the TCPA dataset. The plot reports the top 20 proteomic features ranked according to model-specific importance scores. Feature importance values reflect the relative contribution of each protein to the prediction of therapeutic response. These algorithm-specific rankings were used as input for the consensus-based global feature selection strategy described in the main text.

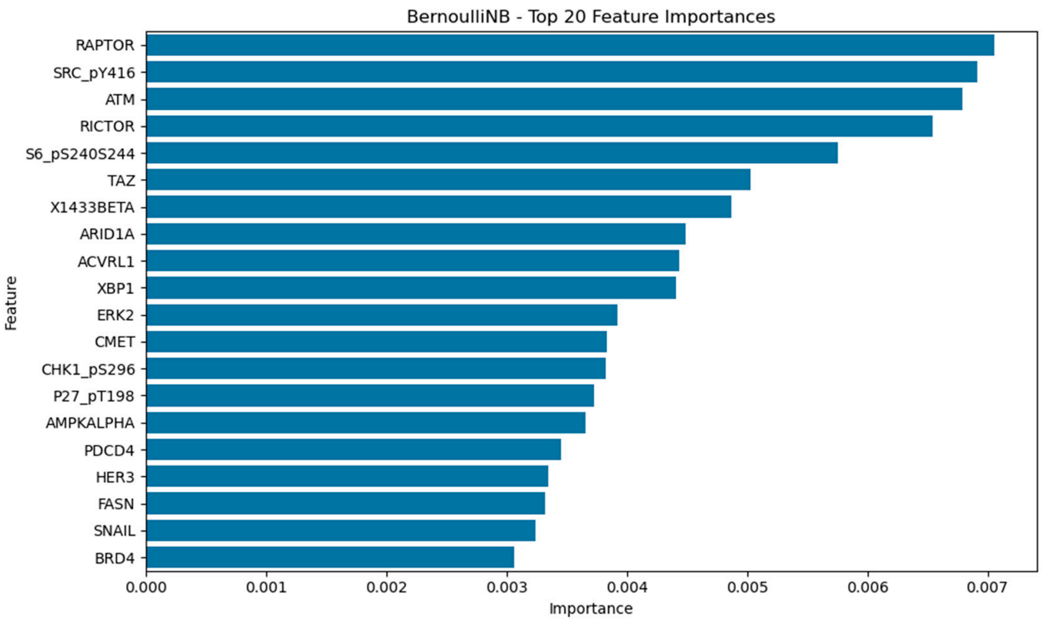

Figure S43. Feature importance analysis for the BernoulliNB classifier applied to the TCPA dataset. The plot reports the top 20 proteomic features ranked according to model-specific importance scores. Feature importance values reflect the relative contribution of each protein to the prediction of therapeutic response. These algorithm-specific rankings were used as input for the consensus-based global feature selection strategy described in the main text.

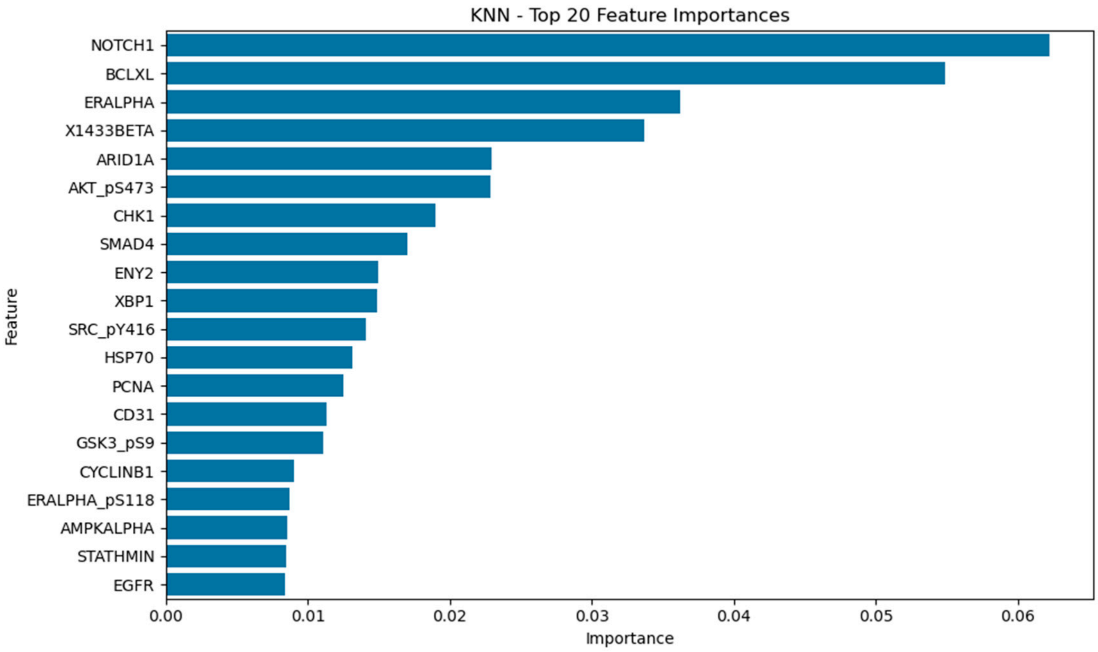

Figure S44. Feature importance analysis for the k-Nearest Neighbors classifier applied to the TCPA dataset. The plot reports the top 20 proteomic features ranked according to model-specific importance scores. Feature importance values reflect the relative contribution of each protein to the prediction of therapeutic response. These algorithm-specific rankings were used as input for the consensus-based global feature selection strategy described in the main text.

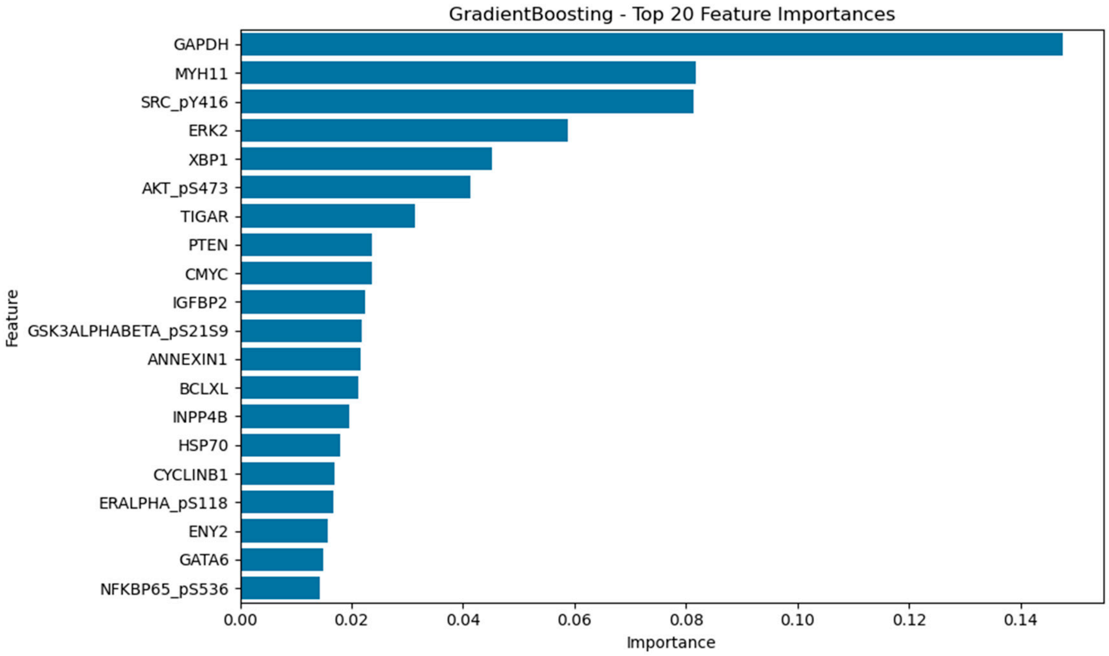

Figure S45. Feature importance analysis for the Gradient Boosting classifier applied to the TCPA dataset. The plot reports the top 20 proteomic features ranked according to model-specific importance scores. Feature importance values reflect the relative contribution of each protein to the prediction of therapeutic response. These algorithm-specific rankings were used as input for the consensus-based global feature selection strategy described in the main text.

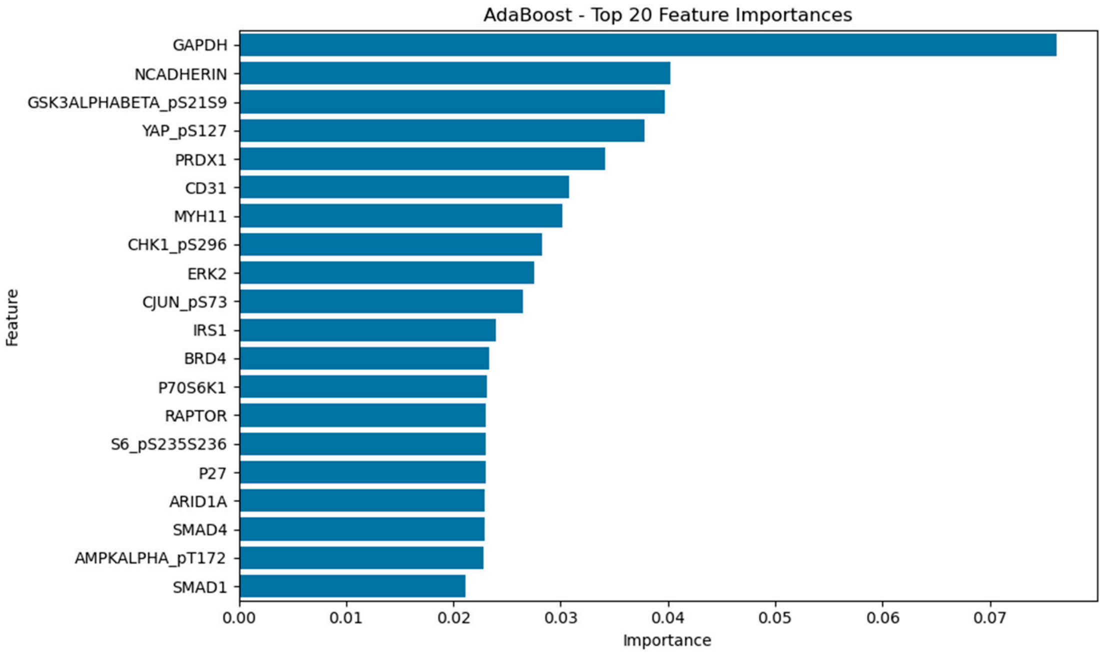

Figure S46. Feature importance analysis for the AdaBoost classifier applied to the TCA dataset. The plot reports the top 20 proteomic features ranked according to model-specific importance scores. Feature importance values reflect the relative contribution of each protein to the prediction of therapeutic response. These algorithm-specific rankings were used as input for the consensus-based global feature selection strategy described in the main text.

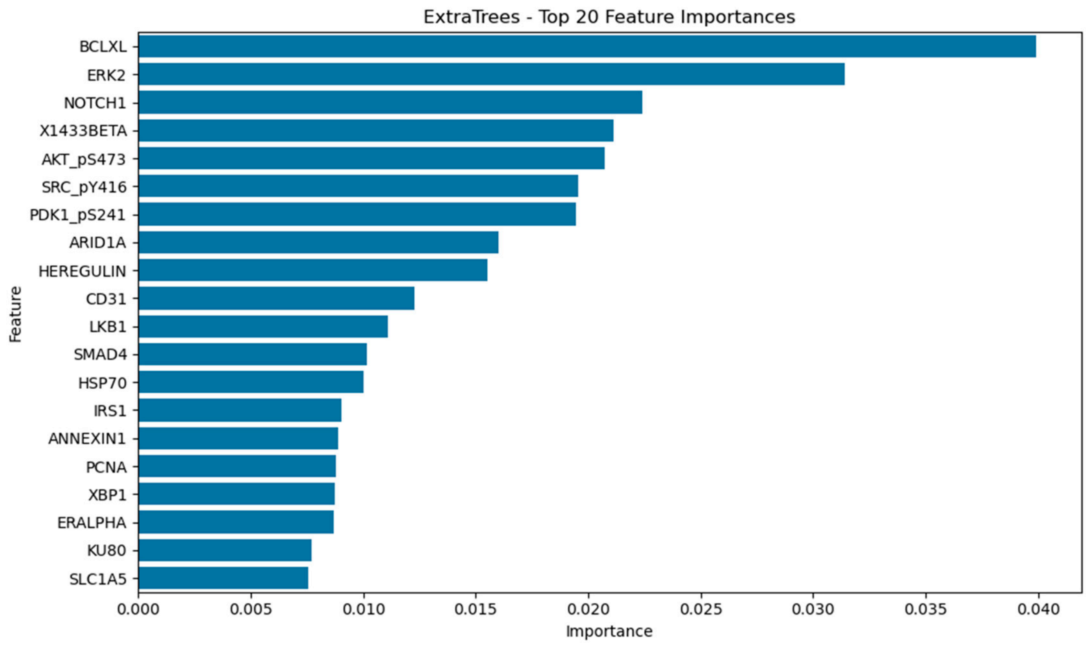

Figure S47. Feature importance analysis for the ExtraTrees classifier applied to the TCGA dataset. The plot reports the top 20 proteomic features ranked according to model-specific importance scores. Feature importance values reflect the relative contribution of each protein to the prediction of therapeutic response. These algorithm-specific rankings were used as input for the consensus-based global feature selection strategy described in the main text.

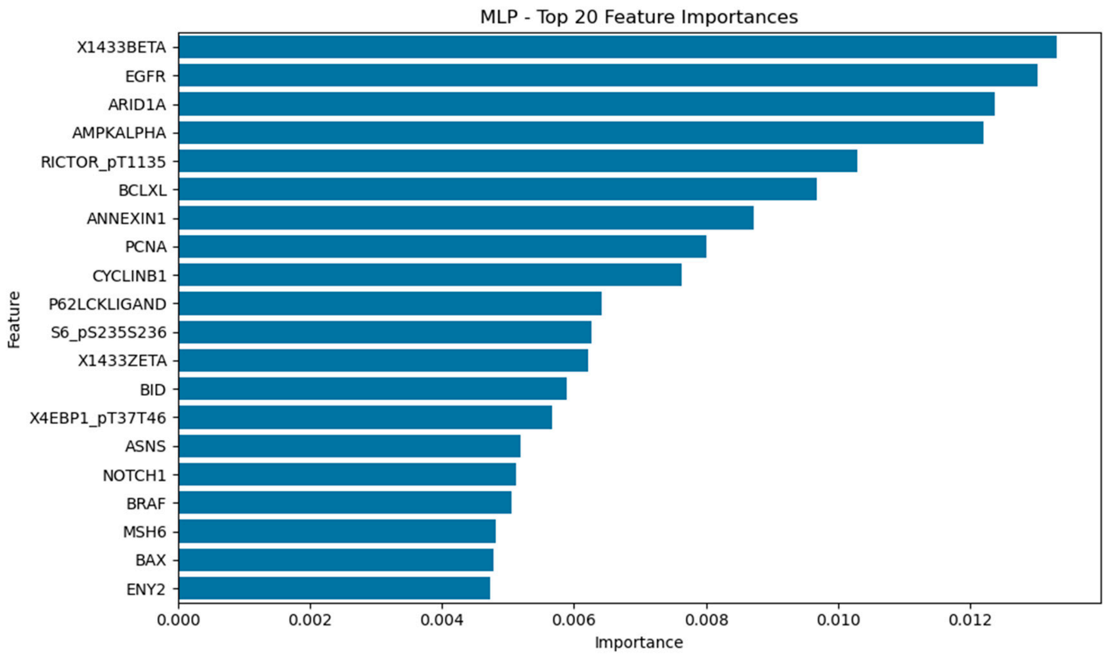

Figure S48. Feature importance analysis for the Multilayer Perceptron classifier applied to the TCGA dataset. The plot reports the top 20 proteomic features ranked according to model-specific importance scores. Feature importance values reflect the relative contribution of each protein to the prediction of therapeutic response. These algorithm-specific rankings were used as input for the consensus-based global feature selection strategy described in the main text.

Table S4 reports the statistical results of the differential expression analysis performed on the top 30 SHAP-selected proteomic features in the TCGA and TCPA cohorts. For each feature, differences between Responders and Non-Responders were assessed using

the non-parametric Mann–Whitney U test. Raw p-values were adjusted for multiple testing using the Benjamini–Hochberg false discovery rate correction. Cliff's delta was calculated to quantify the magnitude and direction of the expression difference between groups. Positive Cliff's delta values indicate higher expression in Responders, whereas negative values indicate higher expression in Non-Responders. Features with FDR-adjusted p-values below 0.05 were considered statistically significant.

Table S4. Differential expression analysis of SHAP-selected proteomic features in TCGA and TCPA cohorts.

Dataset	Feature	Mann-Whitney U	p-value	FDR-adjusted p-value (BH)	Cliff's delta	Direction	Significant at FDR < 0.05
TCGA	CHK1_pS296	3.58E+05	1.04E-06	2.41E-06	0.1703	Higher in Responders	Yes
TCGA	BCLXL	3.24E+05	9.88E-02	1.41E-01	0.0576	Higher in Responders	No
TCGA	HSP70	3.60E+05	5.03E-07	1.26E-06	0.1753	Higher in Responders	Yes
TCGA	SNAIL	3.14E+05	4.33E-01	5.00E-01	0.0273	Higher in Responders	No
TCGA	LCK	2.55E+05	1.96E-06	4.20E-06	-0.1659	Higher in Non-Responders	Yes
TCGA	AMPKALPHA	3.07E+05	9.03E-01	9.03E-01	0.0043	Higher in Responders	No
TCGA	MMP14	2.29E+05	9.66E-12	3.50E-11	-0.2379	Higher in Non-Responders	Yes
TCGA	EphA2_pS897	3.13E+05	2.10E-01	2.62E-01	0.0438	Higher in Responders	No
TCGA	ANNEXIN1	2.29E+05	6.27E-13	3.13E-12	-0.2509	Higher in Non-Responders	Yes
TCGA	RAPTOR	3.93E+05	3.09E-16	1.86E-15	0.2849	Higher in Responders	Yes
TCGA	CKIT	2.42E+05	1.84E-09	5.51E-09	-0.2097	Higher in Non-Responders	Yes
TCGA	S6_pS235S236	2.18E+05	1.21E-16	1.03E-15	-0.2889	Higher in Non-Responders	Yes
TCGA	DRP1	2.07E+05	6.46E-19	1.94E-17	-0.3103	Higher in Non-Responders	Yes
TCGA	KU80	3.14E+05	4.80E-01	5.34E-01	0.0246	Higher in Responders	No
TCGA	IRS1	3.44E+05	3.77E-04	7.07E-04	0.1240	Higher in Responders	Yes
TCGA	FTO	2.13E+05	1.37E-16	1.03E-15	-0.2887	Higher in Non-Responders	Yes
TCGA	ERRalpha	2.28E+05	6.48E-12	2.78E-11	-0.2399	Higher in Non-Responders	Yes
TCGA	YAP_pS127	2.79E+05	1.16E-02	2.04E-02	-0.0881	Higher in Non-Responders	Yes
TCGA	AKT_pS473	2.13E+05	2.05E-18	3.08E-17	-0.3053	Higher in Non-Responders	Yes
TCGA	P62LCKLIGAND	2.95E+05	3.14E-01	3.77E-01	-0.0351	Higher in Non-Responders	No
TCGA	TRAP1	2.79E+05	3.92E-02	6.19E-02	-0.0720	Higher in Non-Responders	No
TCGA	TRANSGLUTAMINASE	3.00E+05	5.45E-01	5.84E-01	-0.0211	Higher in Non-Responders	No
TCGA	FASN	3.51E+05	2.38E-05	4.76E-05	0.1474	Higher in Responders	Yes
TCGA	PKCPANBETA-II_pS660	3.64E+05	5.05E-08	1.38E-07	0.1901	Higher in Responders	Yes
TCGA	P70S6K1	3.00E+05	5.78E-01	5.98E-01	-0.0194	Higher in Non-Responders	No

TCGA	Atg3	3.18E+05	8.69E-02	1.30E-01	0.0598	Higher in Responders	No
TCGA	SRC_pY416	3.22E+05	1.24E-01	1.62E-01	0.0536	Higher in Responders	No
TCGA	4EBP1	2.33E+05	1.05E-11	3.50E-11	-0.2371	Higher in Non-Responders	Yes
TCGA	AR	3.31E+05	1.86E-02	3.10E-02	0.0821	Higher in Responders	Yes
TCGA	MYOSINIIA	2.57E+05	1.06E-01	1.44E-01	0.0590	Higher in Responders	No
TCPA	GAPDH	2.48E+05	1.46E-07	3.36E-07	-0.1835	Higher in Non-Responders	Yes
TCPA	BCLXL	3.06E+05	7.82E-01	7.82E-01	0.0097	Higher in Responders	No
TCPA	X1433BETA	3.50E+05	1.17E-05	2.34E-05	0.1530	Higher in Responders	Yes
TCPA	ARID1A	2.63E+05	1.56E-04	2.75E-04	-0.1320	Higher in Non-Responders	Yes
TCPA	SRC_pY416	3.26E+05	3.17E-02	3.96E-02	0.0750	Higher in Responders	Yes
TCPA	AKT_pS473	2.09E+05	3.26E-19	4.89E-18	-0.3127	Higher in Non-Responders	Yes
TCPA	NOTCH1	2.87E+05	1.16E-01	1.29E-01	-0.0549	Higher in Non-Responders	No
TCPA	AMPKALPHA	2.98E+05	6.17E-01	6.39E-01	-0.0174	Higher in Non-Responders	No
TCPA	ERK2	3.60E+05	9.84E-08	2.46E-07	0.1860	Higher in Responders	Yes
TCPA	RAPTOR	3.91E+05	1.77E-16	1.33E-15	0.2874	Higher in Responders	Yes
TCPA	CYCLINB1	2.89E+05	1.85E-01	1.99E-01	-0.0462	Higher in Non-Responders	No
TCPA	XBP1	2.74E+05	4.81E-03	7.08E-03	-0.0984	Higher in Non-Responders	Yes
TCPA	PDK1_pS241	2.64E+05	1.89E-04	3.15E-04	-0.1303	Higher in Non-Responders	Yes
TCPA	MYH11	2.78E+05	1.72E-02	2.34E-02	-0.0832	Higher in Non-Responders	Yes
TCPA	PRDX1	2.44E+05	1.67E-08	4.56E-08	-0.1969	Higher in Non-Responders	Yes
TCPA	ANNEXIN1	2.22E+05	9.73E-15	5.84E-14	-0.2702	Higher in Non-Responders	Yes
TCPA	GATA6	3.02E+05	2.92E-12	1.25E-11	0.2551	Higher in Responders	Yes
TCPA	ENY2	2.91E+05	1.11E-08	3.32E-08	0.2088	Higher in Responders	Yes
TCPA	S6_pS235S236	2.15E+05	7.05E-17	7.05E-16	-0.2913	Higher in Non-Responders	Yes
TCPA	EGFR	2.26E+05	1.80E-13	9.01E-13	-0.2569	Higher in Non-Responders	Yes
TCPA	TAZ	2.50E+05	3.83E-07	8.21E-07	-0.1772	Higher in Non-Responders	Yes
TCPA	BRAF	3.20E+05	1.10E-01	1.27E-01	0.0558	Higher in Responders	No
TCPA	IRS1	3.34E+05	4.42E-03	6.98E-03	0.0993	Higher in Responders	Yes
TCPA	CMYC	2.84E+05	6.15E-02	7.38E-02	-0.0653	Higher in Non-Responders	No
TCPA	PCNA	2.58E+05	1.58E-05	2.96E-05	-0.1507	Higher in Non-Responders	Yes
TCPA	CKIT	2.40E+05	2.37E-09	8.89E-09	-0.2084	Higher in Non-Responders	Yes
TCPA	MRE11	4.07E+05	1.59E-22	4.78E-21	0.3408	Higher in Responders	Yes

TCPA	GSK3_pS9	2.81E+05	3.14E-02	3.96E-02	-0.0751	Higher in Non-Responders	**Yes**
TCPA	YAP_pS127	2.74E+05	4.95E-03	7.08E-03	-0.0981	Higher in Non-Responders	**Yes**
TCPA	CHK1_pS296	3.65E+05	5.29E-09	1.76E-08	0.2037	Higher in Responders	**Yes**

To evaluate the robustness of the global consensus feature selection strategy, an additional performance-weighted consensus analysis was performed using Script 6. In this analysis, model-specific normalized feature importance scores were weighted according to cross-validated ROC-AUC penalized by fold-to-fold variability, so that features selected by more accurate and stable classifiers contributed more strongly to the final ranking. The resulting performance-weighted top-30 feature panels were compared with the original equal-weight consensus rankings in terms of feature overlap and Jaccard index.

Table S5. Robustness analysis comparing equal-weight and performance-weighted consensus feature rankings. For each cohort, the top-30 features obtained using the original equal-weight consensus ranking were compared with those obtained using a performance-weighted consensus ranking. Model-specific feature importance scores were weighted according to cross-validated ROC-AUC penalized by fold-to-fold variability. Overlap and Jaccard index were computed to assess the stability of the selected proteomic signature.

Dataset	Top-k	Shared features	Overlap	Jaccard index	Equal-weight only	Performance-weighted only
TCGA	30	29/30	96.7%	0.935	4EBP1	LKB1
TCPA	30	30/30	100.0%	1.000	None	None

The performance-weighted consensus analysis showed very high agreement with the original equal-weight ranking. In the TCGA cohort, 29 out of 30 features were shared between the two approaches, corresponding to an overlap of 96.7% and a Jaccard index of 0.935. The only difference was the replacement of 4EBP1 by LKB1 in the performance-weighted ranking. In the TCPA cohort, the two approaches yielded identical top-30 feature panels, with 30 out of 30 shared features and a Jaccard index of 1.000. These results indicate that the selected proteomic signatures are highly robust to the weighting strategy and are not primarily driven by noisy contributions from lower-performing classifiers. The corresponding performance-weighted consensus rankings for the TCGA and TCPA cohorts are shown in Figures S49 and S50, respectively.

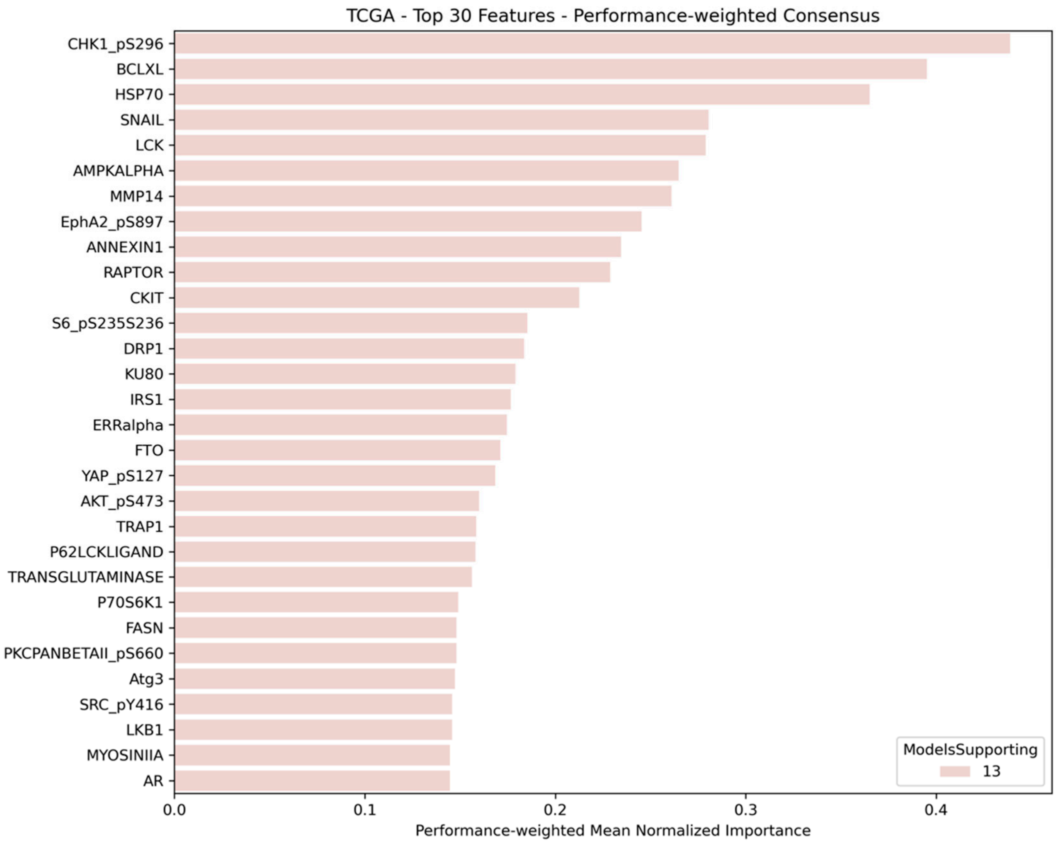

Figure S49. Performance-weighted consensus ranking of the top 30 proteomic features for the TCGA cohort.

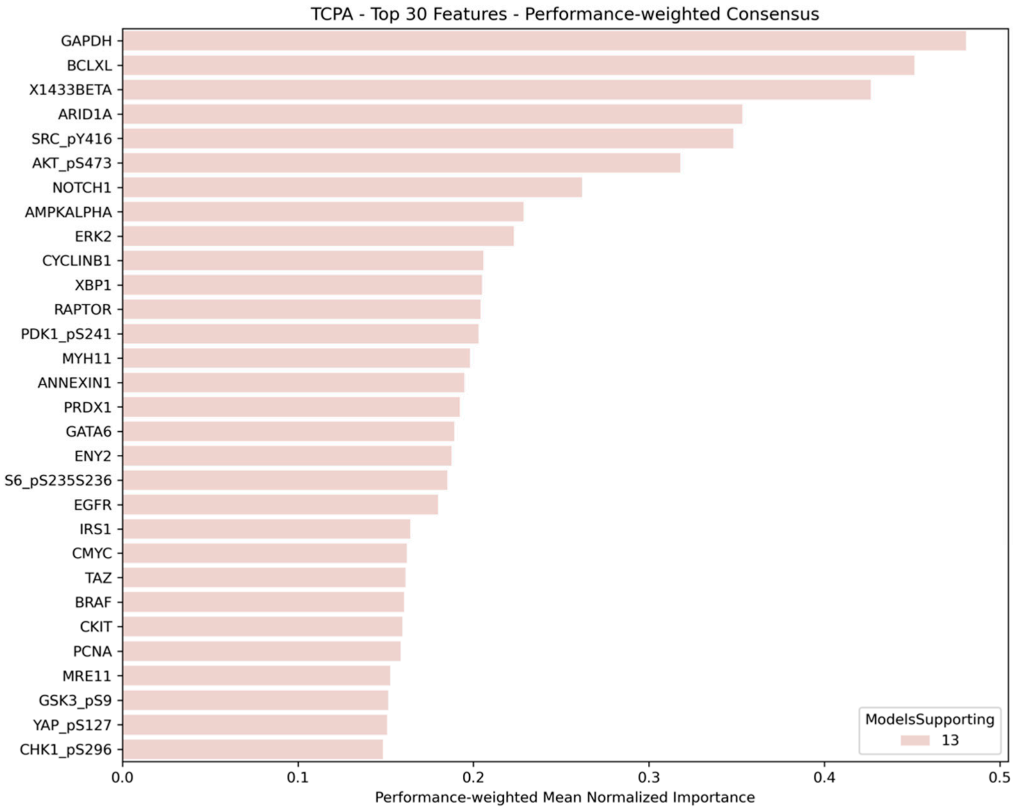

Figure S50. Performance-weighted consensus ranking of the top 30 proteomic features for the TCPA cohort.
